# Moving Forward From COVID-19: Bridging Knowledge Gaps in Maternal Health With a New Conceptual Model

**DOI:** 10.3389/fgwh.2020.586697

**Published:** 2020-11-04

**Authors:** Molly J. Dickens, Jodi L. Pawluski, L. Michael Romero

**Affiliations:** ^1^&Mother Foundation, Berkeley, CA, United States; ^2^Univ Rennes, Inserm, EHESP, Irset (Institut de Recherche en Santé, Environnement et Travail), UMR_S 1085, Rennes, France; ^3^Department of Biology, Tufts University, Medford, MA, United States

**Keywords:** maternal health, stress, maternal mental health, COVID-19, pregnancy, pregnancy physiology, allostasis, reactive scope model

## Abstract

As the world faces the health crisis of a global pandemic—with healthcare protocols in overhaul, and patients and care teams experiencing unprecedented levels of stress and unpredictability—we predict that current knowledge gaps in maternal health will inevitably have a lasting impact on the health of women giving birth now and in the near future. Since we are decades away from closing the knowledge gaps we need filled today, we recommend shifting thinking toward a comprehensive conceptual model that merges knowledge of stress physiology, neurobiology, and pregnancy physiology. The model we present here, the *Maternal Reactive Scope Model*, is an expansion of the Reactive Scope Model built upon the concept of Homeostasis and Allostasis. The model provides a framework to consider pathways and interactions across physiological systems to attribute a physiological basis for considering stress exposure and bridge research gaps on mechanisms to measure or target for treatment. Our intention is to provide an adaptable, heuristic framework for discussion of research considerations and new healthcare models that aim to provide the best care for new mothers during and after the COVID-19 pandemic.

## Introduction

The COVID-19 pandemic has exposed pregnant women to an unprecedented level of stress and unpredictability. Due to the limitations on research addressing the links between stress, human pregnancy physiology, and maternal health, those caring for the pregnant population during this crisis are working with an incomplete model of true risk and potential solutions. As discussed in a recent editorial about COVID-19 and maternal mental health, now is not the time to allow knowledge gaps to hold back care strategies aimed at alleviating stress, and, instead, we need to “proactively develop” these strategies “without delay” (1).

In order to predict vulnerabilities, indicate potential preventions, and facilitate discussion for alternative approaches and considerations for care, we recommend a comprehensive conceptual framework that merges knowledge of stress physiology, neurobiology and pregnancy physiology. Our hope is that a conceptual framework will allow for care considerations that look beyond the current knowledge gaps in maternal health and provide an intellectually satisfying merge between considering both the *adaptive* physiological process of pregnancy, labor, and birth and the increased *susceptibility* to pathology requiring diagnosis, prevention, and treatment.

Here we propose such a framework in the form of the *Maternal Reactive Scope Model*. Our goal in presenting this model is *not* to provide a comprehensive overview of all interconnected physiological pathways and potential health outcomes. Instead, we offer this model as an adaptable, heuristic framework for idea-generation and further discussion.

## Modeling a Body in Balance Yet Pushed to an Extreme

Homeostasis is the biological concept that every physiological *up* is met with a physiological *down* to counterbalance and bring the body back to a steady state. A body out of homeostatic balance is prone to disease. Pregnancy and early postpartum represent a unique homeostatic state in a woman's body— in balance yet pushed to an extreme.

The framework we propose here is built upon the Reactive Scope Model (RSM), a model that considers the balance of maintaining homeostasis in the face of adaptive change (2). The RSM is an expansion of Allostasis, a concept demonstrating how the body maintains stability through change (3). Both the RSM and Allostasis models consider the effects of stress and stressors on the body (both psychological and physical). *Allostatic load* is a key concept to describe the adaptive and maladaptive functions of acute stress and chronic stress (4). Both models describe “*wear-and-tear”* or “*weathering”* as the cost of maintaining responses to counteract stress-related changes in homeostasis and demonstrate how accumulation of these costs put the body at greater risk for entering a disease state (discussed further below).

The RSM factors in the role of physiological mediators that change and respond over a set range and time as they respond to predictable and unpredictable stimuli/stressors. Incorporating the physiological changes associated with pregnancy and postpartum and the critical and natural shifts in physiological mediator ranges, we have adapted the RSM into the Maternal Reactive Scope Model (MRSM). Important to the MRSM is that pregnancy, in and of itself, is *not* considered a disease state, but, through the nature of the physiological changes of pregnancy, the maternal body becomes more *vulnerable* to disease during this time (e.g., hypertension, mood disorders, diabetes, autoimmune diseases, etc.).

Similar to our statement that pregnancy itself is not a disease state, we *do not* consider pregnancy itself to be a stressor or a major contributor to allostatic load or *wear-and-tear*. Rather, the MRSM considers how the stress response system stimulates and/or exacerbates pathological outcomes related to pregnancy, birth, and postpartum.

By providing a framework to conceptualize multiple physiological pathways, the MRSM removes the need to focus on a single physiological system or compartmentalize specific physiological contributions to the risks and pathologies associated with pregnancy. The general understanding of human pregnancy physiology continues to have gaps and will likely have gaps well into the future. Until these knowledge gaps fill, a theoretical framework provides a constructive way to attribute a physiological basis for interventions that demonstrate positive outcomes despite lacking an exact physiological mechanism to measure or target for treatment.

## The Maternal Reactive Scope Model

**Figure 1:**
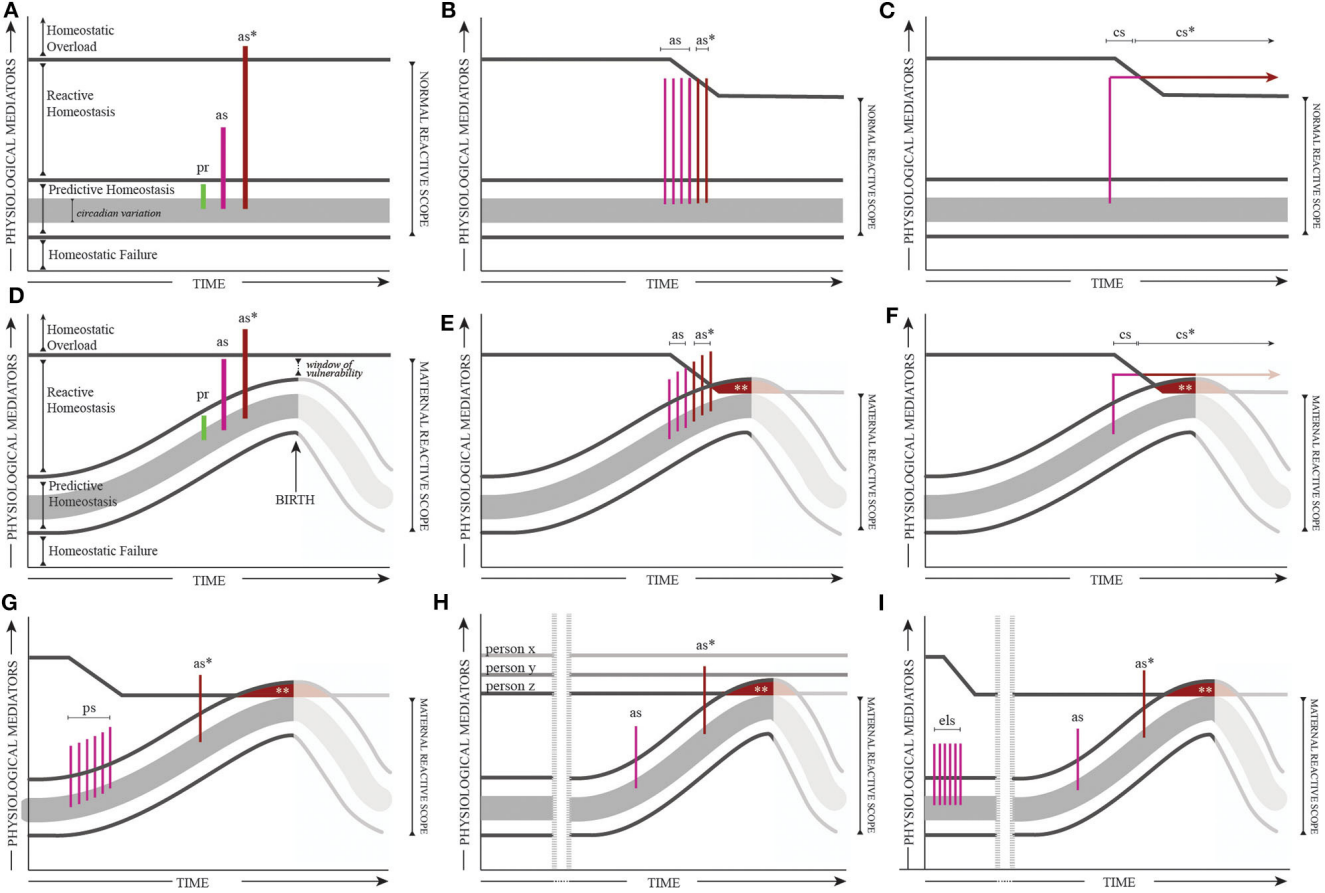
The Reactive Scope Model **(A–C)** and the Maternal Reactive Scope Model **(D–F)** and example modifications of the Maternal Reactive Scope Model **(G–I)**. Acute physiological responses (including responses to stress) represented as “spikes” in the Predictive Homeostasis (pr = predictive response), Reactive Homeostasis (as = adaptive acute stress response), and Homeostatic Overload (as^*^ = maladaptive acute stress response) ranges. The upper threshold between the *reactive scope* and Homeostatic Overload can shift on a short-term or long-term basis in response to stress exposure. Consecutive acute stress responses **(B,E)** and ongoing responses to stress **(C,F)**, lead to “wear-and-tear” that reduces the upper threshold and leads to Homeostatic Overload with additional acute stressors (as^*^) or continuous chronic stress (cs^*^). The *maternal reactive scope* **(D–I)** describes the shift in all ranges of Homeostasis to represent the physiological changes required to sustain pregnancy and prepare for labor and delivery. For simplicity, we demonstrate physiological mediators shifting *up* to represent parameters that *increase* across pregnancy through birth, but recognize that this may be mediator specific. In addition, the postpartum period is represented as a drop in mediator levels, however, the exact nature of these shifts are relatively unknown and may be dependent on the individual (e.g., breastfeeding vs. not breastfeeding) and the mediators studied (e.g., cardiovascular vs. endocrine). Prior research has suggested that the dynamics of the stress response may be buffered during pregnancy, reflected in shorter stress “spikes” later in pregnancy. In these versions of the model, the *maternal reactive scope* is progressively compressed as the lower threshold shifts in response to the mediator requirements of a healthy pregnancy; where this compression peaks is considered a “window of vulnerability” given the increased risk of acute or chronic stress resulting in Homeostatic Overload. A reduced upper threshold for the *maternal reactive scope* can also occur due to **(G)** pregnancy-related stress (ps), **(H)** genetic predisposition, or **(I)** early life stress (els). The compressed *maternal reactive scope* due to a reduced upper threshold and natural increases in the lower threshold can also result in responses or functionality in the Predictive Homeostasis range to become pathological (^**^) and/or previously adaptive acute stress responses (as) crossing into Homeostatic Overload and become maladaptive acute stress response (as^*^) or chronic stress (cs, cs^*^). See Supplemental Material for further breakdown of stress, pregnancy, and the MRSM.

Key to the MRSM, the *physiological mediators* of the y-axis represent any aspect of physiology that regulates homeostasis (Figure 1). These mediators include insulin, cortisol, cardiovascular factors, among others (see Table 1). In the context of the MRSM, these factors change and respond over a set range and across pregnancy, parturition, and postpartum. Four ranges define both adaptive and maladaptive ranges of these mediators: (1) Predictive Homeostasis (2) Reactive Homeostasis, (3) Homeostatic Overload, and (4) Homeostatic Failure (Figure 1A).

**Table 1:** Examples of key physiological systems, relative mediators, the reported shift in mediator levels and/or the role they play in Predictive and Reactive Homeostasis during the perinatal period, and potential health complications for mother, baby or the maternal<>fetal unit when mediators are pushed beyond the upper limit (Homeostatic Overload) or fail to meet the lower limit (Homeostatic Failure).

**Physiological system**	**Physiological mediators**	**Peripartum Predictive/Reactive Homeostasis**	**Peripartum Homeostatic Overload and/or Homeostatic Failure range**
Immune	• Prostaglandin • T-cell activation • Antibody titers • Cytokines	• Pro-inflammatory phases (Support implantation, Parturition) • Anti-inflammatory phase (Maintenance of pregnancy) • Maternal-fetal-placental interactions	• Autoimmune disease • Sensitivity to infectious disease • Preterm birth^*^ • Miscarriage^*^
Endocrine	• HPA ° Glucocorticoids (e.g., cortisol) ° CRH ° Placental CRH (pCRH) ° ACTH • Thyroid • Reproductive • Progesterone • Estrogens • Insulin • Oxytocin • Melatonin • Prolactin	• Cortisol increases 30x nonpregnant concentrations • pCRH becomes dominant driver of maternal HPA • Maternal CRH decreases • HPA responsiveness decreases • Progesterone increases nearly 10x nonpregnant concentrations • Estrogens increase nearly 100x nonpregnant concentrations • Insulin secretion increases 200–250% • Insulin sensitivity decreases up to 50%	• Perinatal mental illness^*^ • Maladaptive fetal HPA development • Preterm birth^*^ • Miscarriage • Insulin resistance • Gestational diabetes mellitus • Preeclampsia • High or low birth weight
Cardiovascular (catecholamines)	• Cardiac output • Stroke volume • Heart rate • Blood pressure • Heart rate variability	• Cardiac output increases 30–50% • Stroke volume increases up to 85 mL (20 weeks gestation) • Heart rate increased (up to 90–100 beats/min) • Systemic vascular resistance decreased by 21% (lowest at 20–24 weeks) • Pulmonary vascular resistance decreased by 34%	• Myocardial infarction • Cardiac muscle breakdown • Hypertension • Preeclampsia
Hematologic and coagulation systems	• White blood cells (WBC) • Red blood cells (RBC) • Erythropoietin • Clotting factors • Fibrinogen	• RBC & WBC counts increase • 30% increase in RBC mass • ~45% increase in plasma volume • Increased erythropoietin production • Hemodilution • Hypercoagulable state	• Anemia • Thromboembolism
Central nervous system	• Neurogenesis • Neurotransmitter concentrations • Cytokines • Neuroendocrine (e.g., Oxytocin) • Neurobehavioral	• Heightened plasticity/malleability of the maternal brain • Increased Oxytocin (maternal bonding)	• Depression • Anxiety • Post-traumatic stress disorder • Attachment disorder

While this is not a comprehensive list, and many links between mediators and complications have been suggested but are not well understood (^*^), the suggested physiological mechanisms and links are intended to be used for generating further discussion of the Maternal Reactive Scope and its potential application.

Predictive Homeostasis is the range of mediators necessary for basic, baseline functionalities that often have a daily circadian rhythm. Mediators will increase yet remain in the Predictive Homeostasis range when responding to predictable challenges (e.g., eating a meal). Reactive Homeostasis includes the range necessary for responding to unpredictable, but *adaptive*, responses (i.e., acute stress response). When mediators exceed the Reactive Homeostasis range, they enter Homeostatic Overload—the mediators themselves become damaging and lead to pathology (labeled as^*^ in Figures 1A,D)—similar to Allostatic Overload in the Allostasis Model. Homeostatic Failure represents the range where mediators are too low to sustain homeostasis.

Put simply, there are specific upper and lower thresholds of “healthy” physiological mediator levels: the lower is the threshold of Homeostatic Failure; the upper is the threshold of Homeostatic Overload. Between the thresholds (the combined range for Predictive and Reactive Homeostasis) is the *normal reactive scope* for an individual - the range required for basic functionality and healthy responses to acute homeostatic perturbations.

During pregnancy, the physiological mechanisms themselves change as the maternal body shifts to prioritize the growth, development, and the birth of the baby [reviewed in (5); represented in Table 1]. Such profound physiological changes often occur only during pregnancy. In the MRSM, the natural changes in physiological parameters across pregnancy are reflected in the increasing requirements for maintaining daily function and predictable challenges as reflected in shifting the Predictive Homeostasis range, thus affecting the lower threshold (altering the *maternal reactive scope*). This shift in the lower threshold is considered normal and required for maintaining a healthy pregnancy.

While normal functioning of physiological systems and the body's ability to react to stimuli in the cases of healthy pregnancies are considered *adaptive*, the physiological requirements to both sustain the health of mom, baby, and the maternal/fetal unit and maintain a homeostatic balance becomes more *precarious* as the *maternal reactive scope* is naturally compressed (see *window of vulnerability* in Figure 1D). Mediator levels that either fail to stay above the lower threshold (inability to maintain Predictive Homeostasis resulting in Homeostatic Failure) or surpass the upper threshold (enter Homeostatic Overload) will likely present as illness, pregnancy complications, and/or developmental issues for the fetus (Table 1). As an example, cortisol and the mediators regulating cortisol concentrations, have non-stress related roles, critical to sustaining and supporting a healthy pregnancy and birth (6)—including: preparing the fetus for the outside world (e.g., thermoregulation, glucose metabolism, lung development), labor/delivery, and activation of mammary glands and milk synthesis. As a result, cortisol concentrations rise throughout pregnancy and peak at the end of the third trimester. While the ties between stress/cortisol physiology and Homeostatic Failure deserve further study, one potential example of Homeostatic Overload and this system may be the rates, risks, and role of stress in perinatal depression as cortisol regulation and stress have been tied to mental health disorders [discussed in (7)].

While the lower threshold naturally shifts with pregnancy, the upper threshold can shift on a short-term or long-term basis in response to stress exposure and affect an individual's *maternal reactive scope* range. Maintaining mediators in the Reactive Homeostasis Range (aka - high allostatic load) due to repeated acute stress without recovery (Figures 1B,E) or prolonged stress (Figures 1C,F) incurs a cost through *wear-and-tear*, resulting in a reduced upper limit. An analogy to demonstrate how *wear-and-tear* affects the body's tolerance for additional physiological pressure is a seesaw balanced with weight on both sides: heavy weights can maintain balance but the seesaw itself experiences more “*wear and tear*,” becoming closer to tipping or breaking, than if lighter weights maintain balance [see (2)].

A compressed *maternal reactive scope* makes the body more vulnerable to stressors as mediators typically operating briefly in the Reactive range more readily cross the upper threshold into Homeostatic Overload (Figure 1E – as^*^). Chronic stress-related shift in upper threshold allows mediators in the Reactive range or Predictive range to cross into Homeostatic Overload (Figures 1E,F – as^*^, cs^*^, ^**^). In pregnancy, chronic stress that exceeds the upper limit and operates in the Homeostatic Overload range may be categorized as “toxic stress,” a term used in fetal/maternal health literature, often in context of negative effects on fetal health and development (8). We predict that life stress (e.g., the global pandemic) poses the most risk toward the end of pregnancy into early postpartum, especially for certain individuals as described below (see Supplemental Material for further breakdown of stress, pregnancy, and the MRSM).

The consideration of physiological mediators in this model is not intended to pinpoint a simplified metric. Rather, the goal here is to provide a starting point for research and clinical conversation. Designing studies to better measure and monitor such mediators will improve our understanding of the balance and healthy range in the context of pregnancy physiology and maternal health. For clinicians, the MRSM is intended as a high-level view, grounded in evidence from physiological research, to consider the natural aspects of physiological changes during pregnancy alongside the increased susceptibility to pathology and the role that stress and stress reduction plays to alleviate or exacerbate health risks. Considering the application suggestions in the following section alongside Table 1 may facilitate both hypothesis generation for future research as well as clinical considerations (see Supplemental Material for further discussion applying the MRSM to brain plasticity and maternal mental illness).

## Applying the Maternal Reactive Scope Model

The MRSM provides a framework of pathological susceptibility in the context of normal physiological changes across pregnancy to facilitate assessment and prediction of individual risk levels.

How the upper threshold of the MRSM is set before pregnancy or altered in response to external stimuli during pregnancy affects which women will experience Homeostatic Overload and when. Importantly, the MRSM relies on an individual responsiveness to a stressor (Figure 1D) Every “spike” in the Reactive range or Overload range indicates an acute stress response that is modifiable and specific to that individual and circumstance. The stress responses themselves are not *all-or-nothing* and require a psychological input to trigger the physiological output. In the context of life stressors and non-infection related COVID-19 stressors, individual differences in resilience and stress reactivity during this time may account for why certain women are affected more markedly than others.

Other individual and circumstantial differences can result in a range of framework permutations. Differences may include how quickly or robustly an individual's mediators respond to stressors, threshold levels between homeostatic ranges, relative steepness of *maternal reactive scope* changes across pregnancy, etc. The integration of these differences could help create a *maternal reactive scope* profile that is unique for each woman and for each pregnancy.

For the sake of simplicity, this initial discussion is restricted to examples where the *maternal reactive scope* is compressed by decreasing the upper threshold and, therefore, the likelihood of entering Homeostatic Overload.

### Stress Exposure During Pregnancy

Often when we think about stress and the impacts during pregnancy, we consider the extreme of traumatic events. In the MRSM, trauma can be reflected as a single stress event stimulating a physiological response that crosses into Homeostatic Overload (spike as^*^ in Figure 1D). Applying this theoretical framework may explain why some women experience negative birth outcomes while others, equally exposed to a traumatic event, appear unaffected. For example, a study compared birth outcomes of women in close proximity to events of the 9/11 terrorist attacks to women who lived five miles away. The researchers found an association between low birth weight, preterm birth and Post-traumatic Stress Disorder (PTSD) diagnosis but no association to proximity (9).

Certain events during pregnancy and birth can also be perceived as *stress* and contribute to increased risk of complications. The *window of vulnerability* predicts that an equivalent stressor might be tolerated early in pregnancy yet cause health problems at the end of pregnancy into postpartum (comparing spike *as* to spike as^*^ in Figure 1D). This prediction may apply to the effects of birth-related stress—in a meta-analysis of maternal stress studies, researchers found that birth-related stress (fear of birth, previous birth trauma) was 2–3x more likely to lead to negative outcomes for baby (low birth weight, preterm birth) than extreme, traumatic events (10).

Even without clear, traumatic stress exposure, the peripartum period is associated with inevitable psychological triggers of the acute stress response—novelty, unpredictability, lack of control. Since a series of small stressors or constant stressors can have a similar effect as a single stressful event and compress the *maternal reactive scope* (see Figures 1D,E), it is important to consider *any* stress during pregnancy but especially during the *window of vulnerability*. In a positive context, improved birth outcomes have been attributed to interventions that likely act by decreasing stress - e.g., benefits of mindfulness, labor support, postpartum support (11).

Chronic stress exposure early in pregnancy can lead to a long-lasting decrease in the upper threshold of the *maternal reactive scope* (Figure 1G), predicting a decreased resilience later. In other words, the MRSM provides a framework for connecting seemingly unrelated psychological impacts and physiological consequences. For example, a study describing connected rates of gestational diabetes mellitus (GDM) and postpartum depression suggested a link between the mental health outcomes and the psychological stress of the GDM diagnosis/associated lifestyle changes (12). Another study found a link between economic downturns and preterm birth rates (13).

Potential physiological and psychological connections are important to consider in the context of COVID-19 as we will likely see effects of the pandemic on maternal health that extend beyond infected patients and beyond individuals currently delivering or preparing to deliver during the crisis. Furthermore, in the context of controlling the spread of COVID-19, acute stress triggers during the peripartum period are ever more present and heightened and many of the traditional avenues for alleviating or limiting stressors during this time (e.g., doulas for labor support, postpartum support at home) may not be options. The MRSM suggests that prevalence and risk of peripartum-related complications are likely to increase, and care strategies aimed at limiting exposure to stressors or decreasing stress directly should be prioritized.

### Genetic Susceptibility to Maternal Complications

For some women, no matter how healthy and stress-free they stay during pregnancy, natural physiological changes of pregnancy will lead to health complications. As an example, genetic susceptibility to perinatal depression likely combines with environmental factors to increase the risk of experiencing a perinatal mental illness (14, 15). Genetic susceptibility is reflected in MRSM as an individual's initial *maternal reactive scope* upper threshold (Figure 1H). Some individuals (e.g., person x, Figure 1H) have a naturally high *maternal reactive scope* and are thus more resilient. In contrast, for some individuals (e.g., person z, Figure 1H), the levels of physiological mediators required to maintain Predictive or Reactive Homeostasis and support pregnancy, naturally cross into Homeostatic Overload at some point in pregnancy and lead to illness and/or complications for mother, baby or the maternal/fetal unit. Other individuals (e.g., person y, Figure 1H) may have an intermediate upper threshold such that they will not necessarily experience pregnancy-related health issues but will be less resilient to stress exposure during pregnancy.

Variation in genetic susceptibility may explain why certain individuals are more vulnerable or resilient to stressors during pregnancy. In the context of pandemic-related life stress, genetically susceptible women may experience the effects of stress more acutely or earlier in their pregnancy.

### Life Stress Effects on Maternal Health

Continuous or chronic stress exposure prior to gestation can lead to *wear-and-tear* and a reduced threshold to Homeostatic Overload, making women more vulnerable to illness and complications during pregnancy when the *maternal reactive scope* is further compressed.

An example of sustained and chronic stressors prior to pregnancy that may affect pregnancy outcomes is the growing evidence that institutionalized racism underlies the racial disparities in maternal and infant morbidity and mortality (16). The concept of *weathering* in Black Americans, especially Black women, describes a physiological vulnerability to disease that is directly tied to racism-based stress exposure and measure-able as a difference in allostatic load in individuals and populations (17). *Weathering* has been applied to examine the racial disparity in birth outcomes (18) and can be conceptualized with the MRSM as a reduced upper threshold such that women more readily experience ill effects of Homeostatic Overload during/after pregnancy due to chronic stress exposure prior to pregnancy (Figure 1I).

In times of pandemic crisis and restructuring of prenatal healthcare, we must consider all sources of stress to better care for the most impacted and at-risk individuals and communities.

## Conclusions

We are living through a global pandemic that has forced us to re-think many of our policies in maternal health while working with and around large knowledge gaps. As discussed in a prior review, uninfected pregnant and postpartum individuals will likely face increased negative health outcomes during and immediately following the COVID-19 pandemic (1). Our hope in presenting a conceptual framework is to bridge current knowledge gaps and start a dialogue of alternative strategies to discuss, describe, and consider the connections between pregnancy physiology, stress, and maternal health. In addition, we anticipate that researchers will add their own permutations to the model to expand it to prediction and application.

Specific stress triggers during pregnancy (related and unrelated to the pandemic) may be hard to directly measure and monitor given the individual nature of the stress response system. The MRSM provides a lens for higher-level consideration of both the cumulative effects of stress exposure and the implications of seemingly “smaller” stressors, especially during more sensitive windows of time. Wide-spread uncertainty, financial instability, racism, and increased demands on the healthcare system are classic stressors insofar as they contain key psychological elements (novelty, unpredictability, lack of control) that could trigger the physiological stress response. In addition, during the global pandemic, many of the traditional avenues for alleviating or limiting stressors may not be options. For healthcare teams adapting and building new models of care, we hope the MRSM highlights the need to discuss and consider stress and stress-buffering in prenatal and postpartum care and the importance of decreasing stress exposure whenever possible.

While individual women will have different *maternal reactive scope* ranges and react differently to individual stressors (i.e., more or less resilient), the added stressors associated with the COVID-19 pandemic could have global maternal health implications, especially in already at-risk individuals and communities.

As we move through and beyond this pandemic, we hope the MRSM will aid the progress in advancing maternal health on a global scale by providing a physiological framework for optimizing research, prioritizing considerations of stress exposure, and inspiring the development and adoption of new strategies for prediction and personalized care.

## Data Availability Statement

The original contributions presented in the study are included in the article/Supplementary Material, further inquiries can be directed to the corresponding author/s.

## Author Contributions

MD and LR contributed to initial conceptualization of the model. JP contributed case study examination. MD drafted the initial manuscript. All authors contributed to exploration and discussion of model application and further definitions and provided critical revision of intellectual content.

## Conflict of Interest

The authors declare that the research was conducted in the absence of any commercial or financial relationships that could be construed as a potential conflict of interest.
