# Supplementary material for: Moving Forward From COVID-19: Bridging Knowledge Gaps in Maternal Health With a New Conceptual Model
Source: Front Glob Womens Health. 2020 Nov 4;1:586697. doi: 10.3389/fgwh.2020.586697 (PMC8593985; doi:10.3389/fgwh.2020.586697)
Supplement: Supplementary file 1 [file Data_Sheet_1.pdf]

Supplemental material

Case study: Maternal Reactive Scope Model applied to COVID-19, maternal brain, and maternal mental illness.

During pregnancy and the postpartum period, there are remarkable changes in the brain of the parent, most significantly in the mother, with research showing that in humans there is an overall decrease in brain volume of 4% [1] prior to parturition which returns back to preconception size during the postpartum period. More recently research in women has shown the specificity of these structural changes remarking that a number of brain areas important for aspects of maternal care-giving behaviors also show structural changes, generally decreasing, during the transition to parenting [2]. A growing body of research in human and most animal models has documented the neurophysiology, neuroendocrine and neurobehavioral factors that contribute to heightened plasticity or malleability of the maternal brain. This increased brain plasticity with the transition to motherhood is normal and is linked to the rapid onset of maternal care of offspring [2,3]. In the context of the Maternal Reactive Scope Model (MRSM), we can think of the maternal brain as showing rather 'extreme' levels of plasticity, speculated to be far greater than at other times in a woman's life but still within the normal reactive scope during this time. This 'extreme' brain plasticity is healthy and important, not only for bonding to the neonate, but for other aspects of maternal care such as the onset of lactation [4]. But what happens when this system gets pushed too far and the range of brain plasticity ends up falling outside of the 'healthy range'? The MRSM would predict a disease state. And, indeed, this seems quite plausible.

Although we don't know nearly enough about the neuroscience of maternal mental illnesses, by applying the MRSM to changes in the maternal brain, one would speculate that genetic susceptibility and/or exposure to repeated or chronic stress (such as a pandemic, previous experience with mental illness, severe stress, and a negative birth experience) would more readily push the healthy plasticity in the maternal brain beyond the upper limit of the maternal reactive scope and into a pathological Homeostatic Overload state resulting in a postpartum mental illness.

For example, the amygdala, an important relay center for communication-related to maternal behaviors [5,6] undergoes normal changes associated with the transition to parenting (compressed *maternal reactive scope*). However, the addition of extreme stimulation in response to cues associated with inter partner violence [7] or a traumatic birth, could push such changes in the amygdala into Homeostatic Overload range, change neurocircuitry and lead to postpartum depression or postpartum post-traumatic stress disorder.

While this discussion currently remains in the context of theoretical speculation, applying the MRSM to the changes in the brain during pregnancy and postpartum — a system in a healthy state but pushed to its maximum — allows for a different view on how one needs only an additional 'stressor' to put that system into a dysfunctional state. This something that is relevant today with the effect of COVID-19 on labor & delivery and postpartum support. So many new mothers are faced with traumatic birthing experiences, fear of contracting COVID-19, and lack

of support postpartum. Now more than ever we need the MRSM*:* a conceptual framework that will aid in predicting risk and implementing policies and treatments that will benefit the mental health of the mother, child, and family.

REFERENCES:

1. Oatridge A, Holdcroft A, Saeed N, Hajnal JV, Puri BK, Fusi L, et al. Change in brain size during and after pregnancy: study in healthy women and women with preeclampsia. AJNR Am J Neuroradiol. 2002;23: 19–26.

2. Hoekzema E, Barba-Müller E, Pozzobon C, Picado M, Lucco F, García-García D, et al. Pregnancy leads to long-lasting changes in human brain structure. Nat Neurosci. 2017;20: 287–296.

3. Hoekzema E, Tamnes CK, Berns P, Barba-Müller E, Pozzobon C, Picado M, et al. Becoming a mother entails anatomical changes in the ventral striatum of the human brain that facilitate its responsiveness to offspring cues. Psychoneuroendocrinology. 2020;112: 104507.

4. Slattery DA, Neumann ID. No stress please! Mechanisms of stress hyporesponsiveness of the maternal brain. J Physiol. 2008;586: 377–385.

5. Feldman R. The adaptive human parental brain: implications for children's social development. Trends Neurosci. 2015;38: 387–399.

6. Pawluski JL, Lonstein JS, Fleming AS. The Neurobiology of Postpartum Anxiety and Depression. Trends Neurosci. 2017;40: 106–120.

7. Roos A, Fouche J-P, Stein DJ. Brain network connectivity in women exposed to intimate partner violence: a graph theory analysis study. Brain Imaging Behav. 2017;11: 1629–1639.
