# Supplementary material for: Moving Forward From COVID-19: Bridging Knowledge Gaps in Maternal Health With a New Conceptual Model
Source: Front Glob Womens Health. 2020 Nov 4;1:586697. doi: 10.3389/fgwh.2020.586697 (PMC8593985; doi:10.3389/fgwh.2020.586697)
Supplement: Supplementary file 2 [file Presentation_1.pdf]

Stress, pregnancy, and the Maternal Reactive Scope Model

Molly J. Dickens, PhD
Jodi L. Pawluski, PhD
L. Michael Romero, PhD

Supplemental to:
Dickens MJ, Pawluski JL and Romero LM (2020) Moving Forward From COVID-19: Bridging Knowledge Gaps in Maternal Health With a New Conceptual Model.
Front. Glob. Womens Health 1:586697. doi: 10.3389/fgwh.2020.586697

Dickens MJ, Pawluski JL and Romero LM (2020) Moving Forward From COVID-19: Bridging Knowledge Gaps in Maternal Health With a New Conceptual Model. Front. Glob. Womens Health 1:586697. doi: 10.3389/fgwh.2020.586697

Contents

What is "stress"?

- A mini physiology lesson
- Let's get theoretical – *The Reactive Scope Model*

Why is pregnancy a special case?

- The *Maternal Reactive Scope Model*
- Pregnancy is NOT a disease state BUT…
- Stress and pregnancy

Now what?

- How to consider "stress" in pregnancy.
- **CASE STUDY– Maternal brain plasticity and perinatal mental illness**

Appendix

- List of physiological mechanisms, the known and unknown.
- Example: the Hypothalamic-Pituitary-Adrenal axis

What is “Stress”

From: Dickens MJ, Pawluski JL and Romero LM (2020) Moving Forward From COVID-19: Bridging Knowledge Gaps in Maternal Health With a New Conceptual Model. Front. Glob. Womens Health 1:586697. doi: 10.3389/fgwh.2020.586697

Stress = the physiological response to a ***stressor***.

Also called a ***stress response***.

Stressor = an external stimuli that initiates a ***stress response****.

*yes, even stress physiologists have a hard time defining "stress" without circular logic.

From: Dickens MJ, Pawluski JL and Romero LM (2020) Moving Forward From COVID-19: Bridging Knowledge Gaps in Maternal Health With a New Conceptual Model. Front. Glob. Womens Health 1:586697. doi: 10.3389/fgwh.2020.586697

Classic acute stress response =

escape the predator.

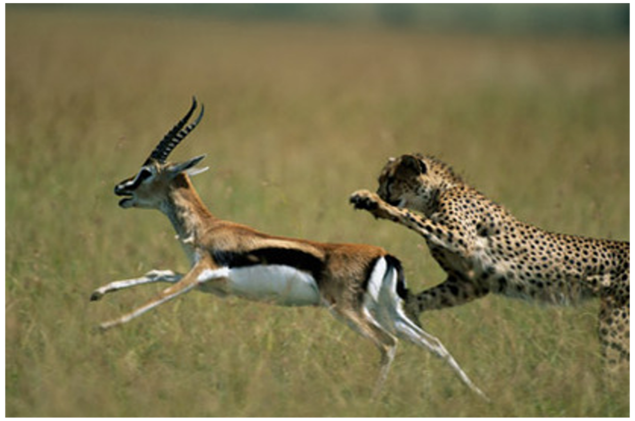

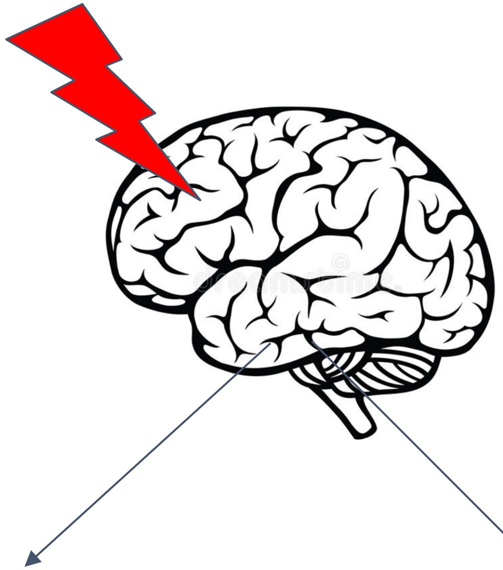

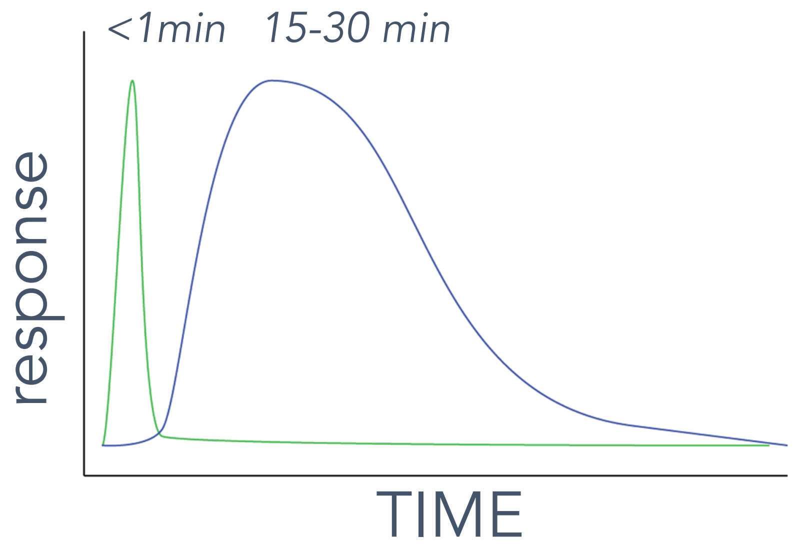

Rapid Fight-or-Flight Response
(epinephrine, norepinephrine)

- Cardiac output
- Vigilance
- Energy mobilization

Slower Glucocorticoid Response
(cortisol)

- Metabolism
- Immune function
- Reproduction
- Behavior

From: Dickens MJ, Pawluski JL and Romero LM (2020) Moving Forward From COVID-19: Bridging Knowledge Gaps in Maternal Health With a New Conceptual Model. Front. Glob. Womens Health 1:586697. doi: 10.3389/fgwh.2020.586697

Some things to keep in mind…

A stress response is (most often) the ***physiological manifestation*** of a ***psychological input.***

- If you don't *perceive* the input as a stressor, you won't *mount* a stress response.

Not all stress responses are created equal.

- Different pathways regulate ***how much*** you physiologically respond to a stressor.
  - Physiological changes/differences can affect how *your body* physically responds to a specific stressor.
- The physiological response can be moderated.
  - Individual/contextual differences in ***perception of the severity*** of the stressor determine ***your*** individual response.
  - It's not all or nothing -- For example, you can mount a Fight-or-Flight response without a cortisol response.

From: Dickens MJ, Pawluski JL and Romero LM (2020) Moving Forward From COVID-19: Bridging Knowledge Gaps in Maternal Health With a New Conceptual Model. Front. Glob. Womens Health 1:586697. doi: 10.3389/fgwh.2020.586697

Let's get theoretical…

Stress in the context of homeostasis.

From: Dickens MJ, Pawluski JL and Romero LM (2020) Moving Forward From COVID-19: Bridging Knowledge Gaps in Maternal Health With a New Conceptual Model. Front. Glob. Womens Health 1:586697. doi: 10.3389/fgwh.2020.586697

The body works to stay in balance.

This is called ***homeostasis***.

Every physiological ***up*** is met with a physiological (or behavioral) ***down*** to counterbalance and return to body to a **steady state**.

A system out of balance = disease/pathology.

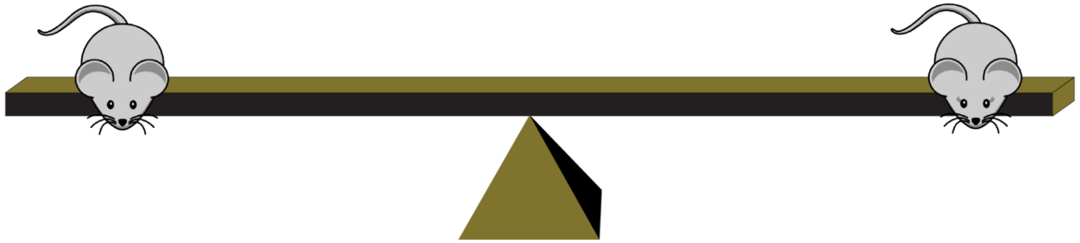

From: Dickens MJ, Pawluski JL and Romero LM (2020) Moving Forward From COVID-19: Bridging Knowledge Gaps in Maternal Health With a New Conceptual Model. Front. Glob. Womens Health 1:586697. doi: 10.3389/fgwh.2020.586697

Stress (*the stress response*) tests the boundaries of homeostasis BUT…

The stress response is an ***adaptive*** response. It is ***beneficial*** *for survival.*

The *acute stress response* has its own pathways and mechanisms to *return to homeostasis* and get the body back to its steady state.

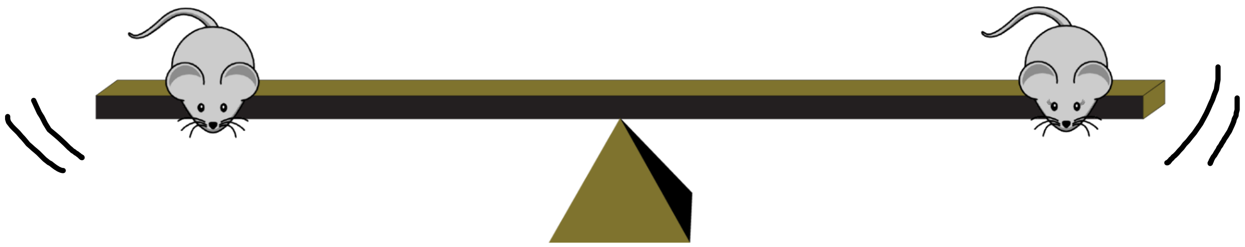

From: Dickens MJ, Pawluski JL and Romero LM (2020) Moving Forward From COVID-19: Bridging Knowledge Gaps in Maternal Health With a New Conceptual Model. Front. Glob. Womens Health 1:586697. doi: 10.3389/fgwh.2020.586697

Here’s another way to think about homeostasis and stress…

*Physiological mediators**
regulate processes in the body and respond to maintain homeostasis
(e.g. insulin, cortisol, cardiovascular factors, etc.)

They change and respond over a set range and across time.

This is the basis for the
Reactive Scope Model

Reactive Scope Model

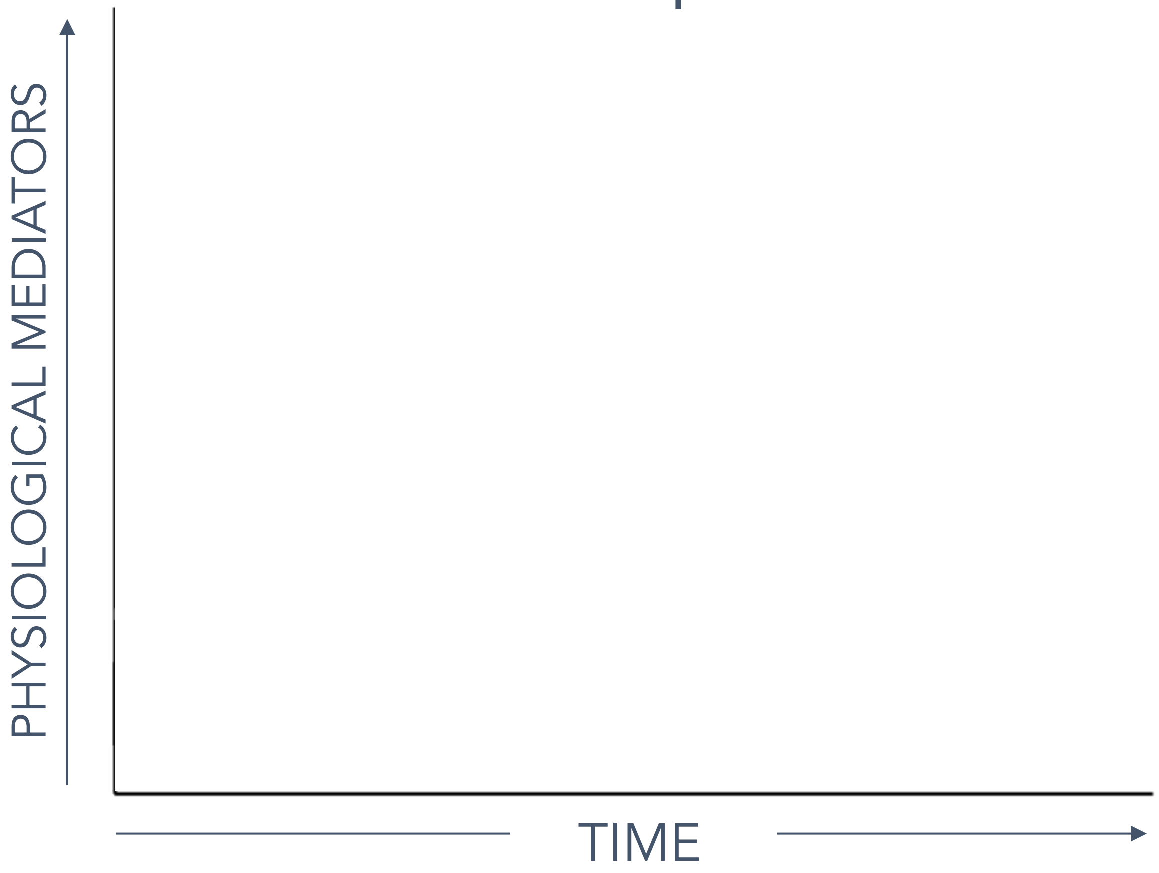

**See appendix for examples of physiological mediators.*

From: Dickens MJ, Pawluski JL and Romero LM (2020) Moving Forward From COVID-19: Bridging Knowledge Gaps in Maternal Health With a New Conceptual Model. Front. Glob. Womens Health 1:586697. doi: 10.3389/fgwh.2020.586697

Physiological mediators change and respond over a set range and across time…

From: Dickens MJ, Pawluski JL and Romero LM (2020) Moving Forward From COVID-19: Bridging Knowledge Gaps in Maternal Health With a New Conceptual Model. Front. Glob. Womens Health 1:586697. doi: 10.3389/fgwh.2020.586697

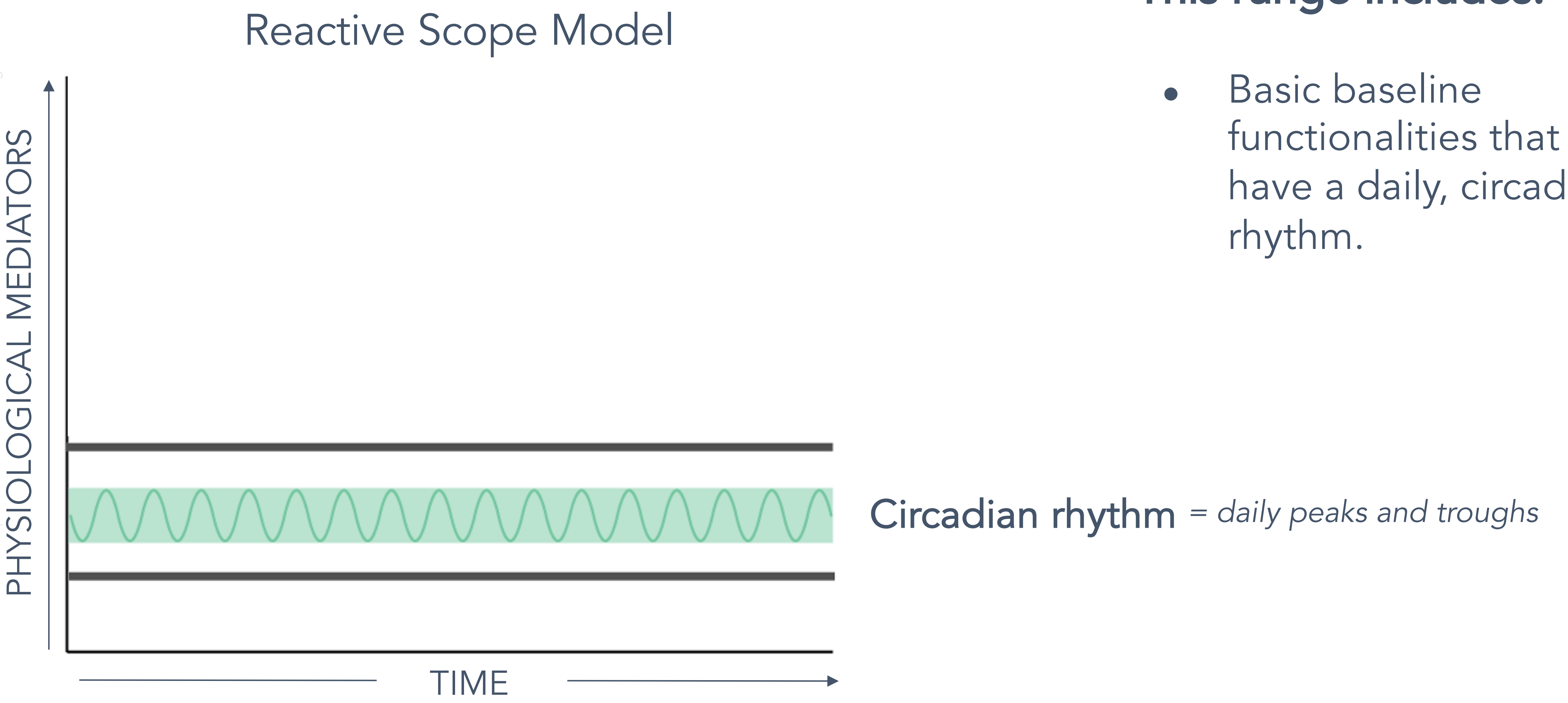

This range includes:

- Basic baseline functionalities that often have a daily, circadian rhythm.

Physiological mediators change and respond over a set range and across time…

Reactive Scope Model

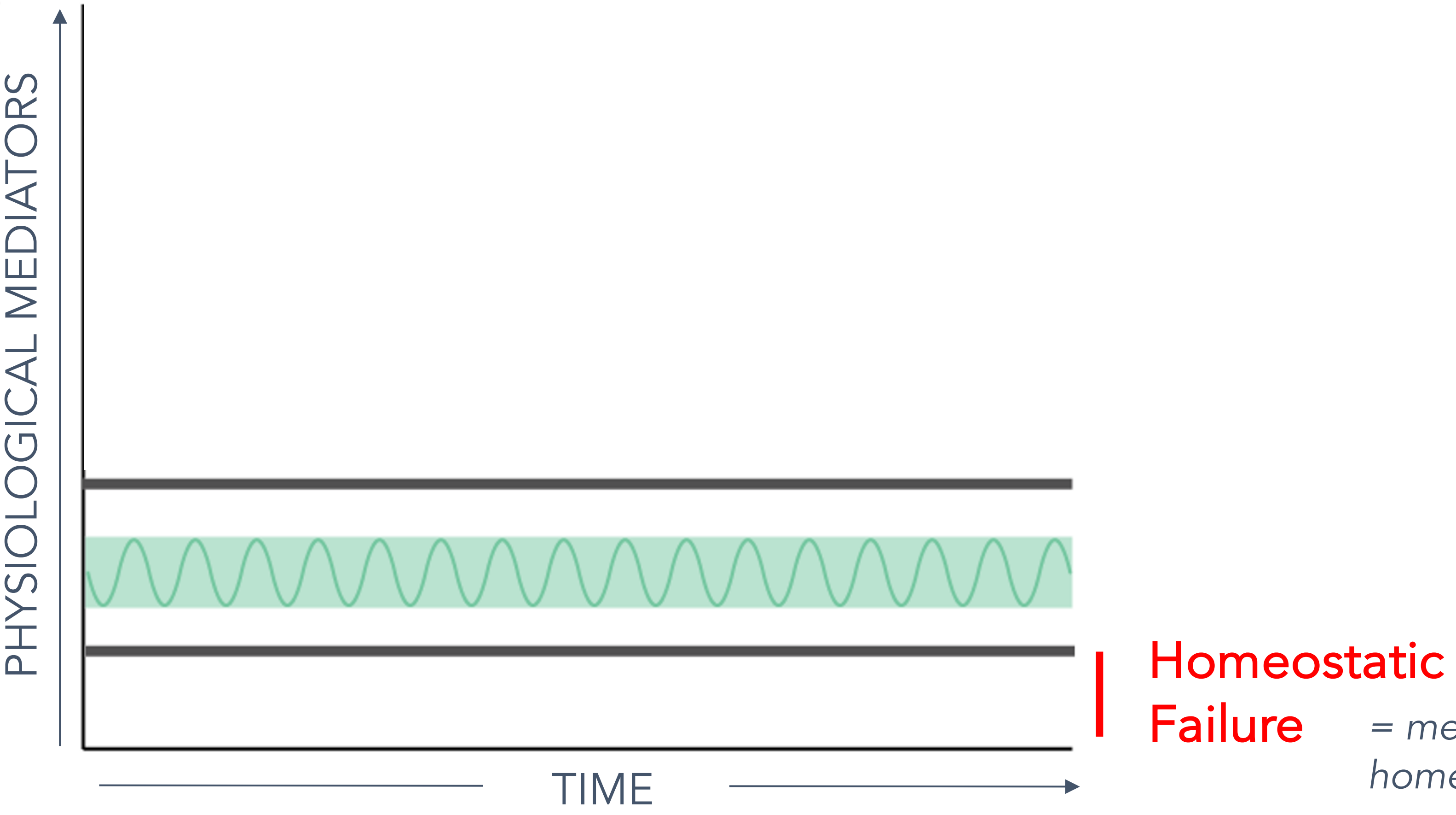

= *mediator levels are too low to sustain basic homeostatic functionalities*

From: Dickens MJ, Pawluski JL and Romero LM (2020) Moving Forward From COVID-19: Bridging Knowledge Gaps in Maternal Health With a New Conceptual Model. Front. Glob. Womens Health 1:586697. doi: 10.3389/fgwh.2020.586697

This range includes:

- Basic baseline functionalities that often have a daily, circadian rhythm.

Physiological mediators change and respond over a set range and across time…

Reactive Scope Model

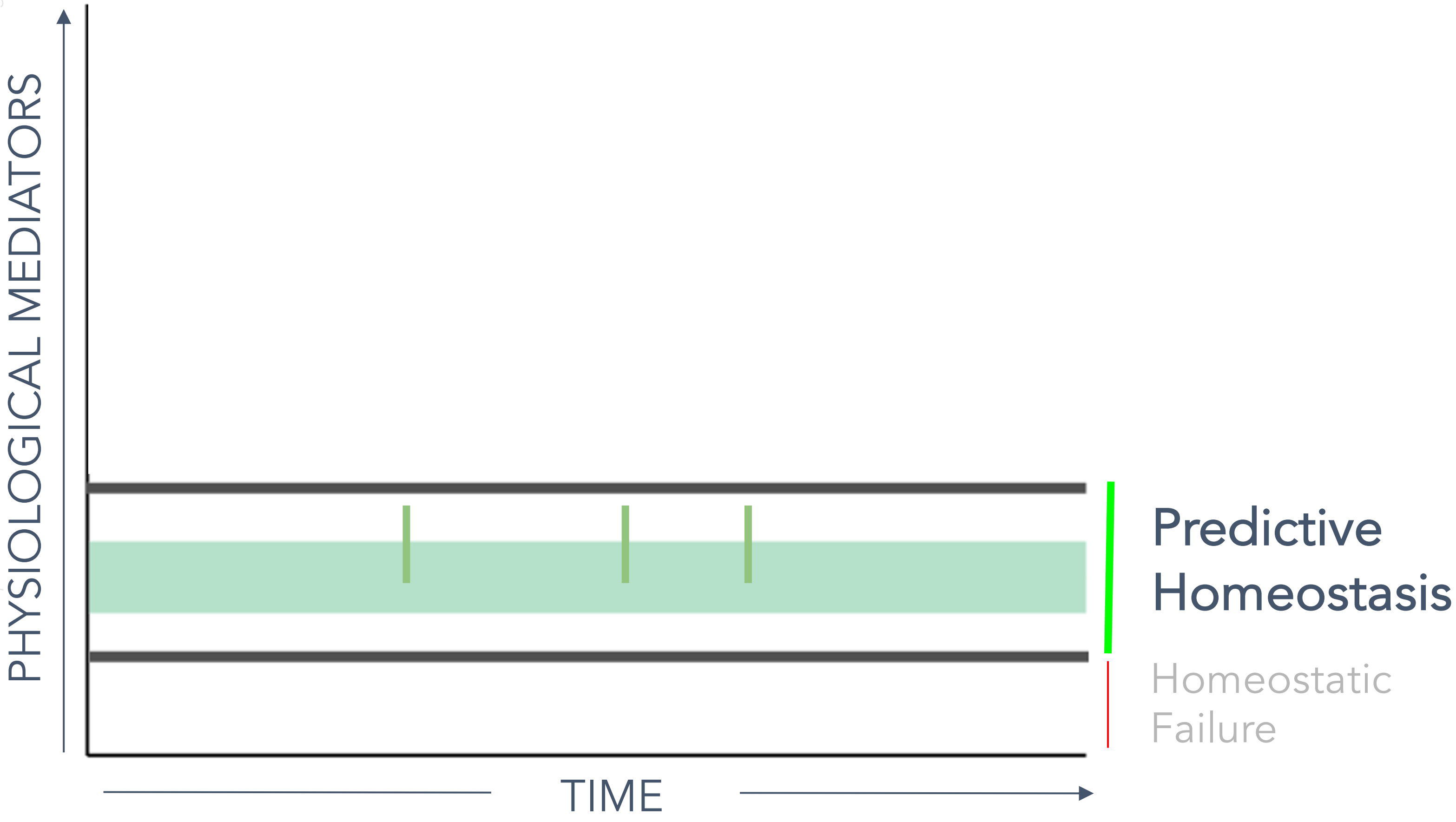

= *mediator levels temporarily elevated but return to baseline when elevated levels are no longer required.*

This range includes:

- Basic baseline functionalities that often have a daily, circadian rhythm
- Necessary and predictable responses (e.g. eating a meal)

From: Dickens MJ, Pawluski JL and Romero LM (2020) Moving Forward From COVID-19: Bridging Knowledge Gaps in Maternal Health With a New Conceptual Model. Front. Glob. Womens Health 1:586697. doi: 10.3389/fgwh.2020.586697

Physiological mediators change and respond over a set range and across time…

Reactive Scope Model

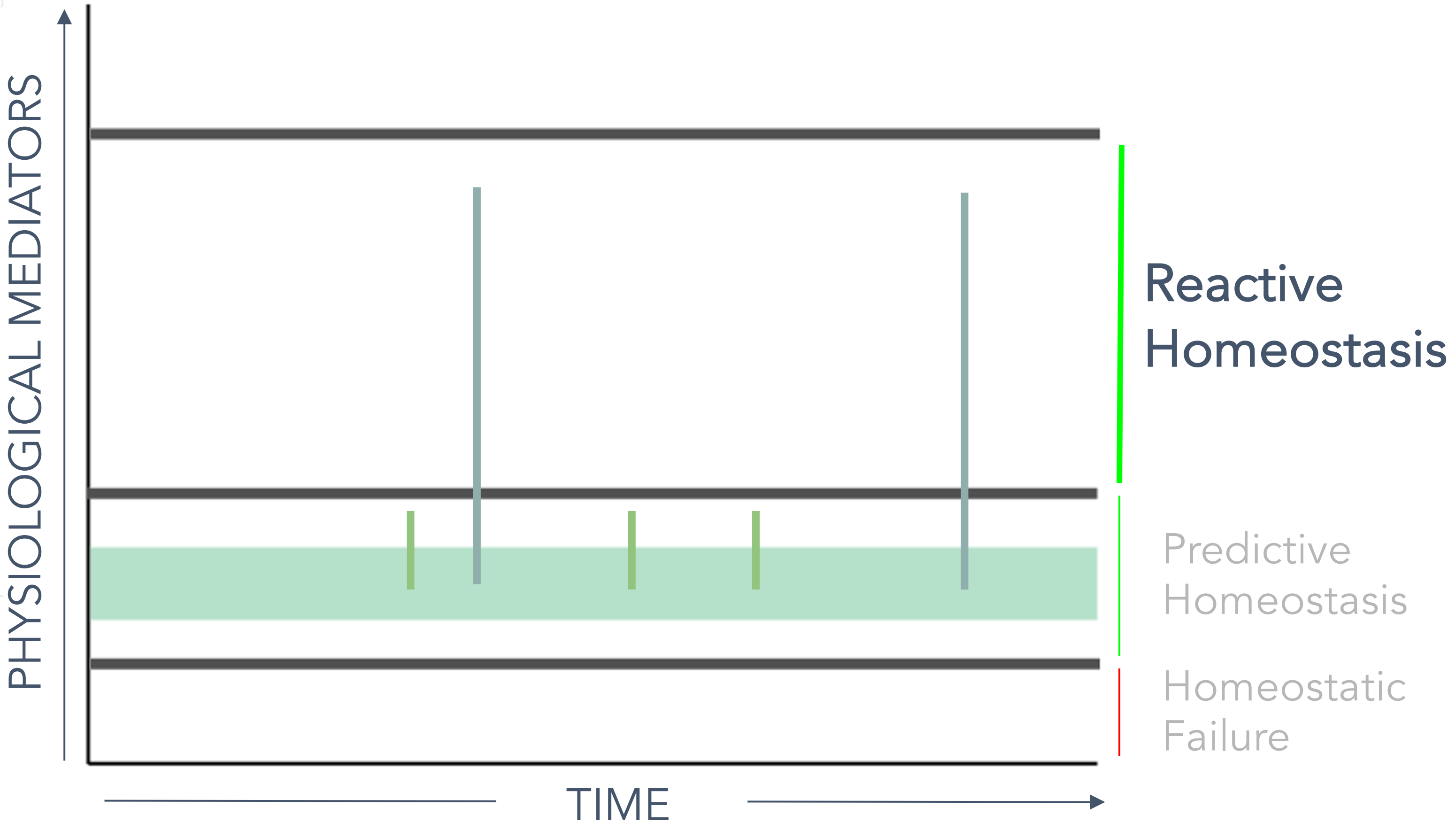

From: Dickens MJ, Pawluski JL and Romero LM (2020) Moving Forward From COVID-19: Bridging Knowledge Gaps in Maternal Health With a New Conceptual Model. Front. Glob. Womens Health 1:586697. doi: 10.3389/fgwh.2020.586697

This range includes:

- Basic baseline functionalities that often have a daily, circadian rhythm
- Necessary and predictable response (e.g. eating a meal)
- Unpredictable but still adaptive responses (*acute stress response*)

Physiological mediators change and respond over a set range and across time…

Reactive Scope Model

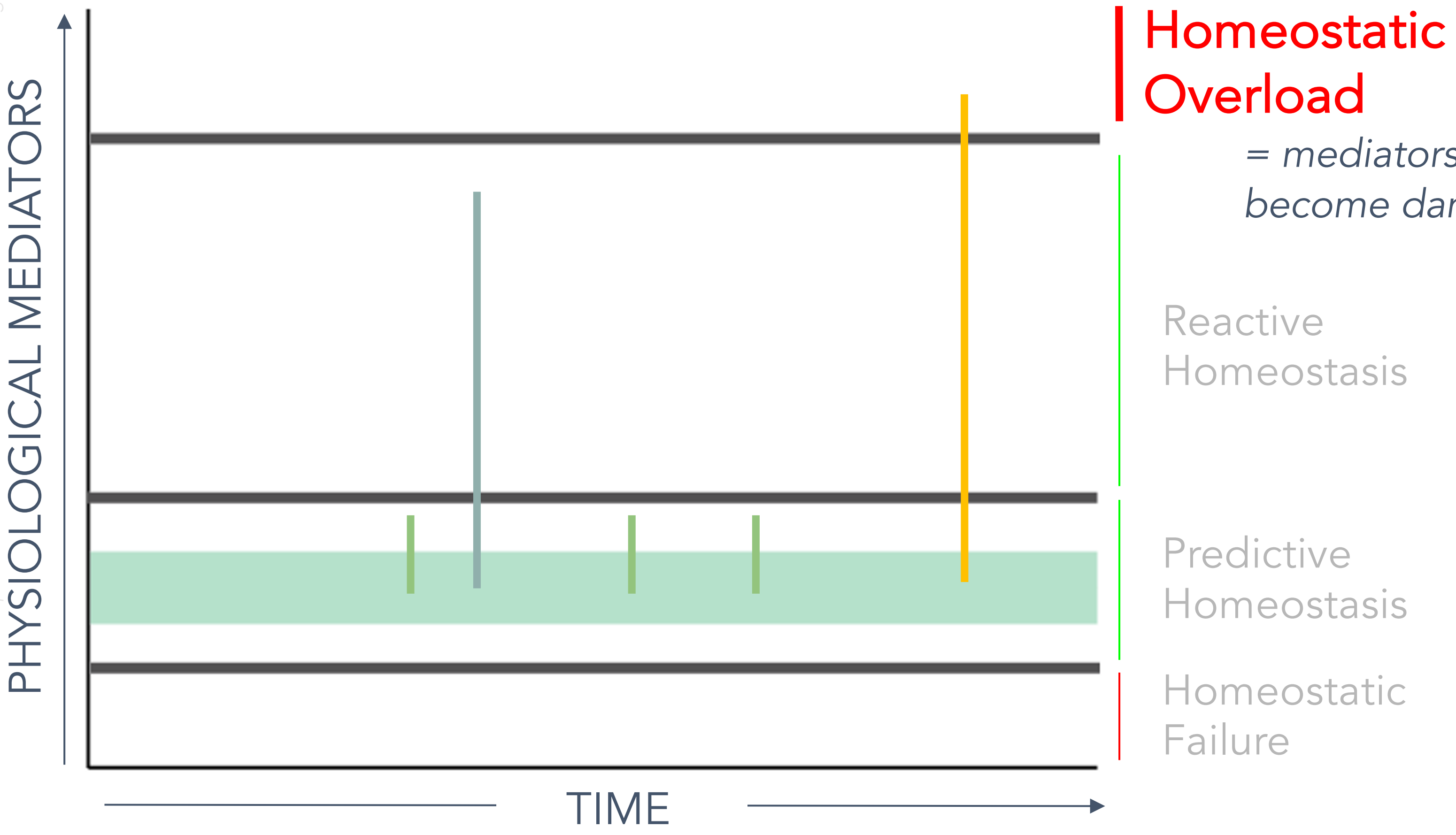

From: Dickens MJ, Pawluski JL and Romero LM (2020) Moving Forward From COVID-19: Bridging Knowledge Gaps in Maternal Health With a New Conceptual Model. Front. Glob. Womens Health 1:586697. doi: 10.3389/fgwh.2020.586697

This range includes:

- Basic baseline functionalities that often … aily, circadian
- Necessary and predictable response (e.g. eating a meal)
- Unpredictable but still adaptive responses (*acute stress response*)
- Levels exceeding what a system can recover from without affecting health.

Physiological mediators change and respond over a set range and across time…

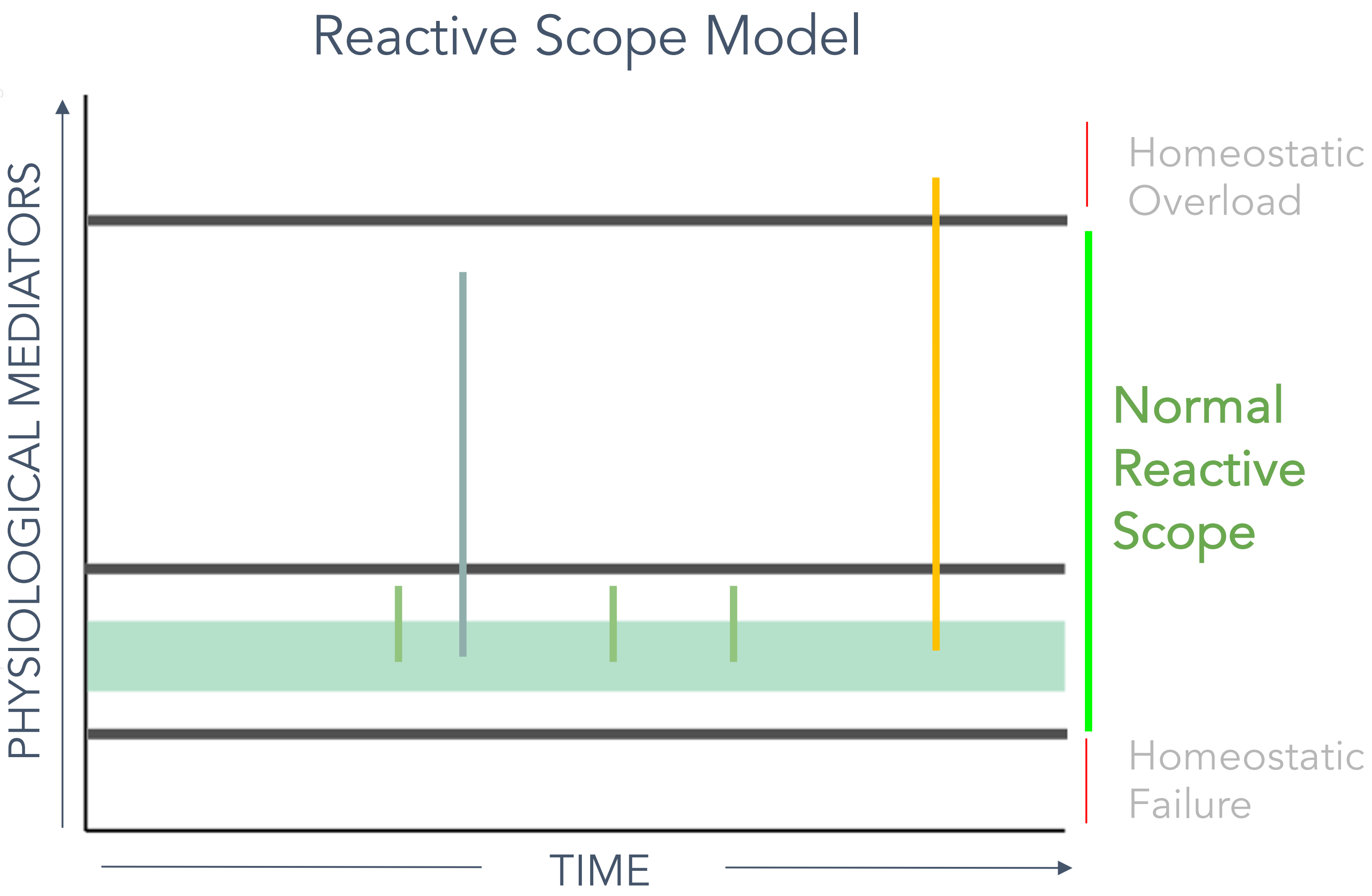

From: Dickens MJ, Pawluski JL and Romero LM (2020) Moving Forward From COVID-19: Bridging Knowledge Gaps in Maternal Health With a New Conceptual Model. Front. Glob. Womens Health 1:586697. doi: 10.3389/fgwh.2020.586697

This range includes:

- Basic baseline functionalities that often have a daily, circadian rhythm
- Necessary and predictable response (e.g. eating a meal)
- Unpredictable but still adaptive responses (*acute stress response*)
- Levels exceeding what a system can recover from without affecting health

Physiological mediators change and respond over a set range and across time…

Reactive Scope Model

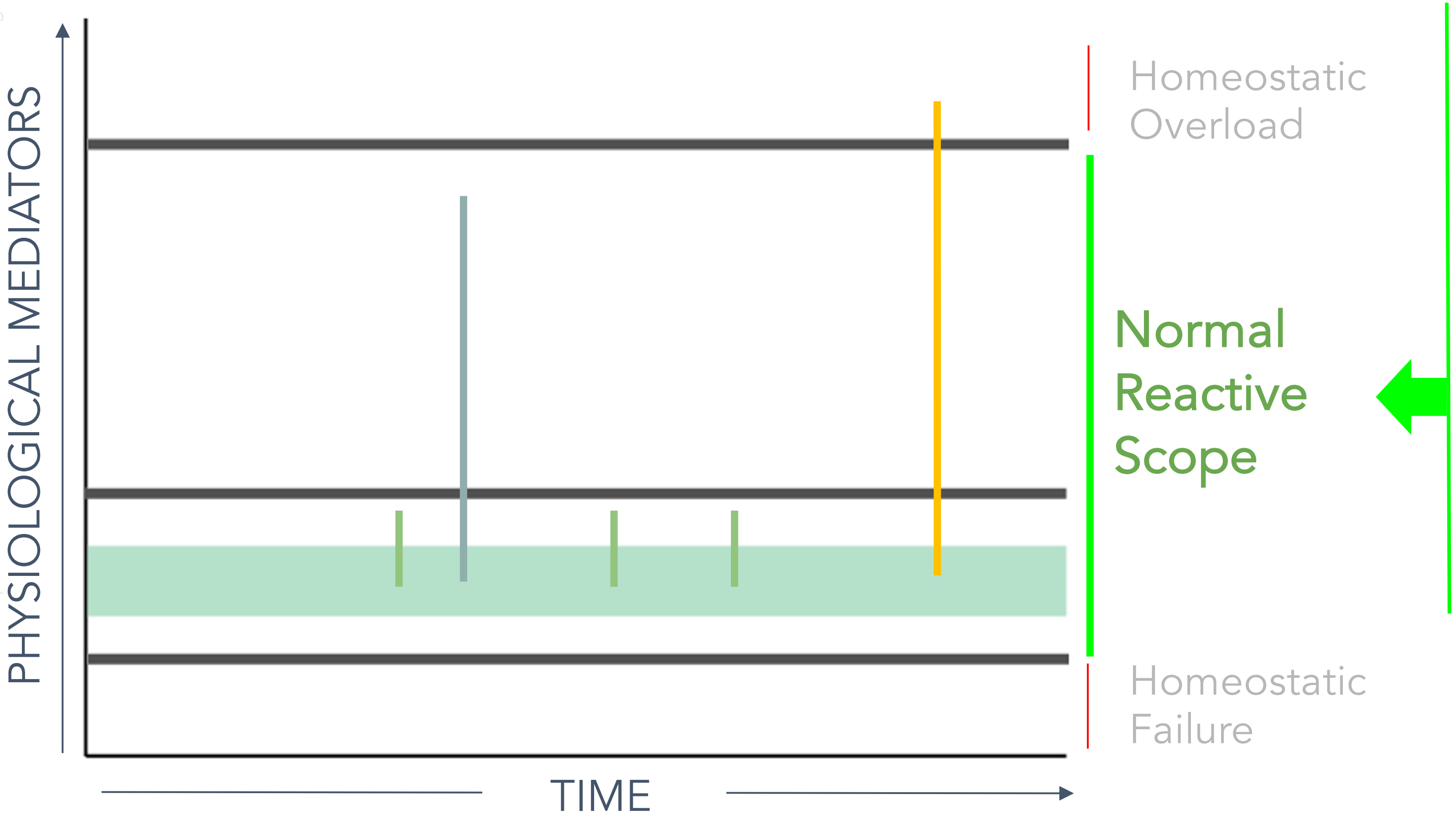

= mediator range for basic functionality and healthy response to acute homeostatic perturbations.

From: Dickens MJ, Pawluski JL and Romero LM (2020) Moving Forward From COVID-19: Bridging Knowledge Gaps in Maternal Health With a New Conceptual Model. Front. Glob. Womens Health 1:586697. doi: 10.3389/fgwh.2020.586697

The body does not normally respond in the *Homeostatic Overload* range, unless...

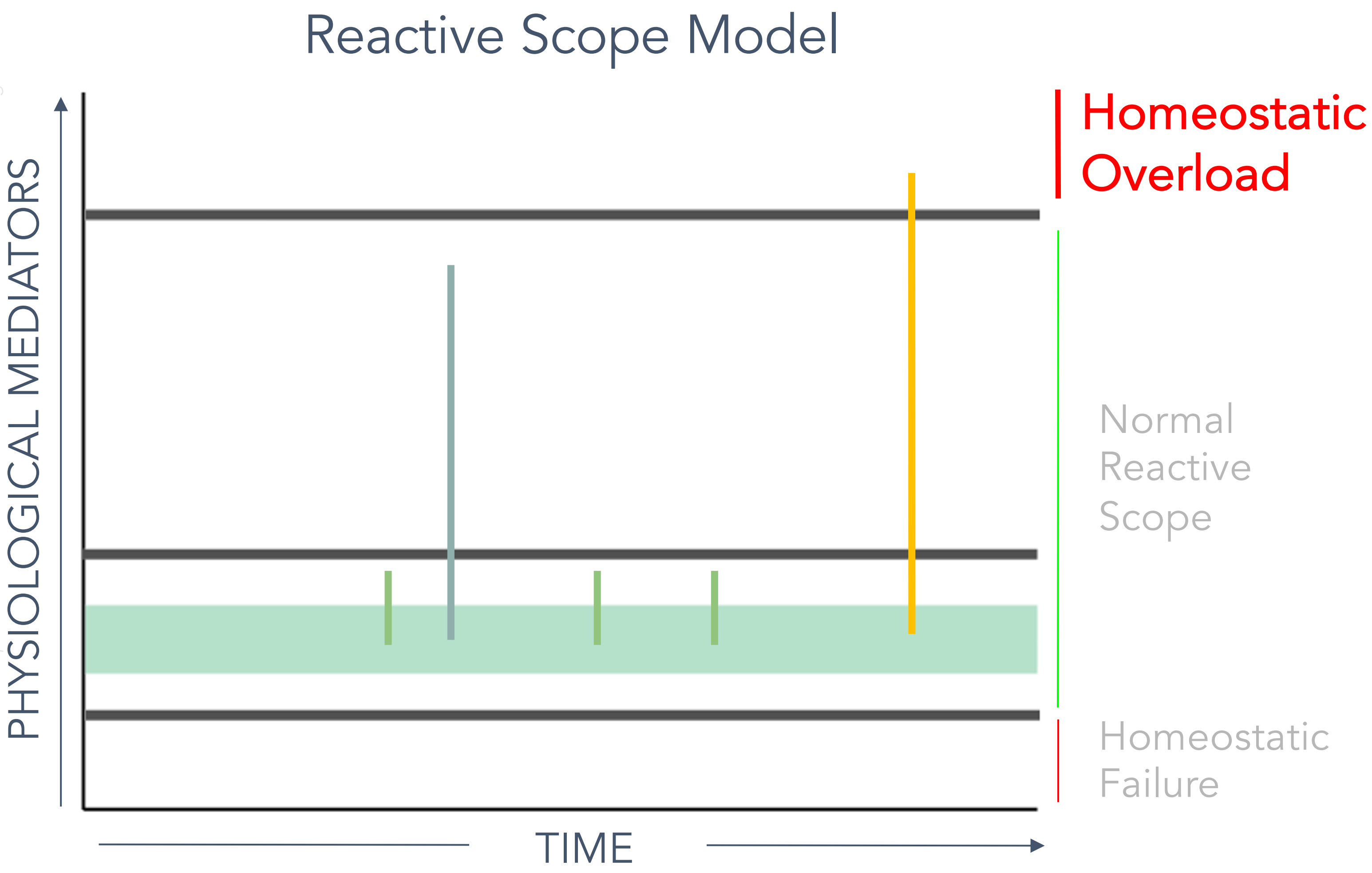

From: *Dickens MJ, Pawluski JL and Romero LM (2020) Moving Forward From COVID-19: Bridging Knowledge Gaps in Maternal Health With a New Conceptual Model. Front. Glob. Womens Health 1:586697. doi: 10.3389/fgwh.2020.586697*

The body does not normally respond in the *Homeostatic Overload* range, unless…

Reactive Scope Model

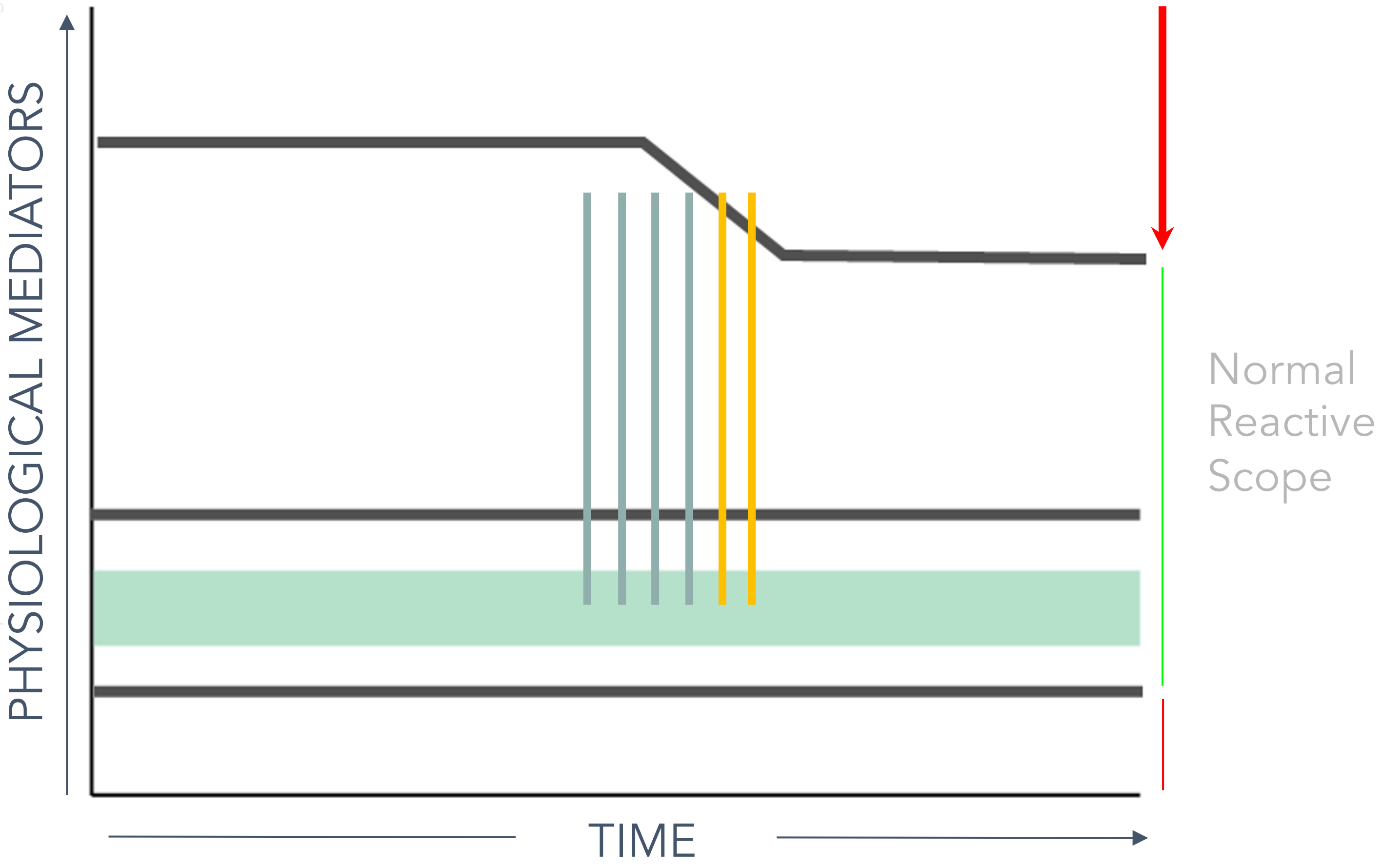

From: Dickens MJ, Pawluski JL and Romero LM (2020) Moving Forward From COVID-19: Bridging Knowledge Gaps in Maternal Health With a New Conceptual Model. Front. Glob. Womens Health 1:586697. doi: 10.3389/fgwh.2020.586697

…the physiological mediators are pushed ***beyond normal functionality*** and overcompensate to maintain balance.

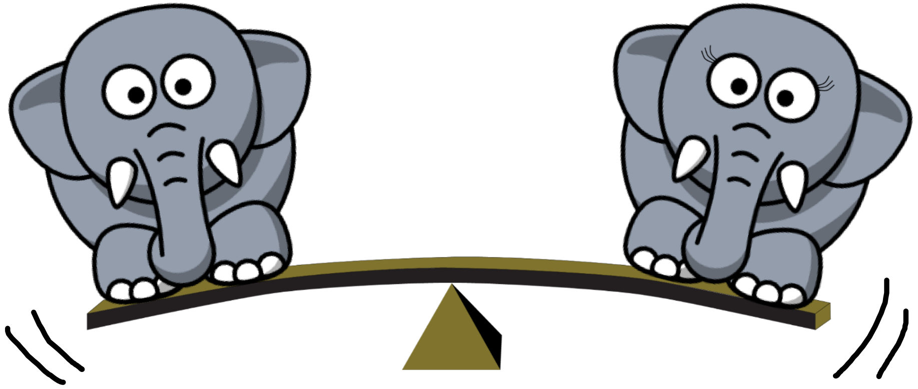

There is a cost to mediators operating at high levels without returning to set point.

The body does not normally respond in the *Homeostatic Overload* range, unless…

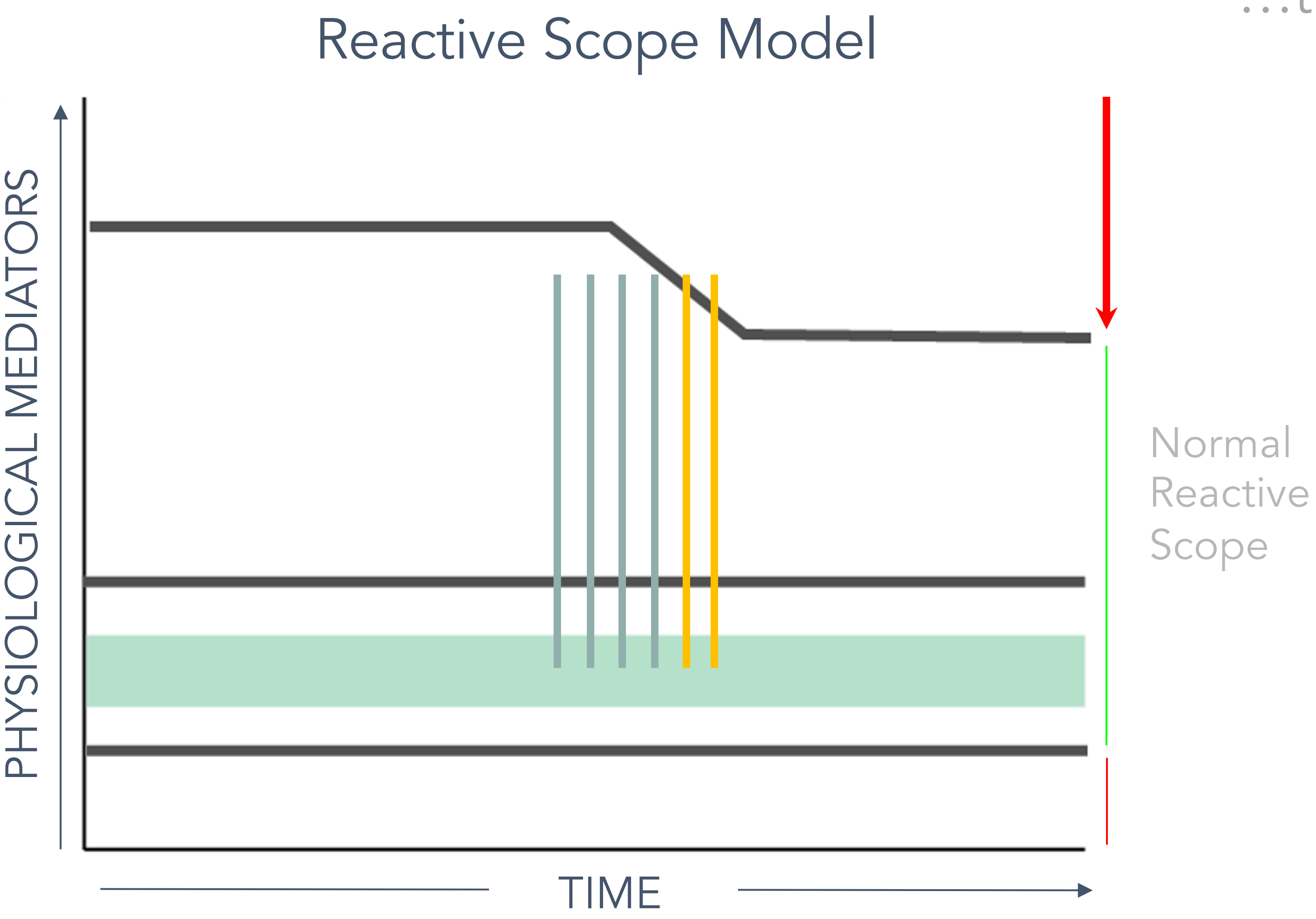

From: Dickens MJ, Pawluski JL and Romero LM (2020) Moving Forward From COVID-19: Bridging Knowledge Gaps in Maternal Health With a New Conceptual Model. Front. Glob. Womens Health 1:586697. doi: 10.3389/fgwh.2020.586697

…the physiological mediators are pushed ***beyond normal functionality*** and overcompensate to maintain balance.

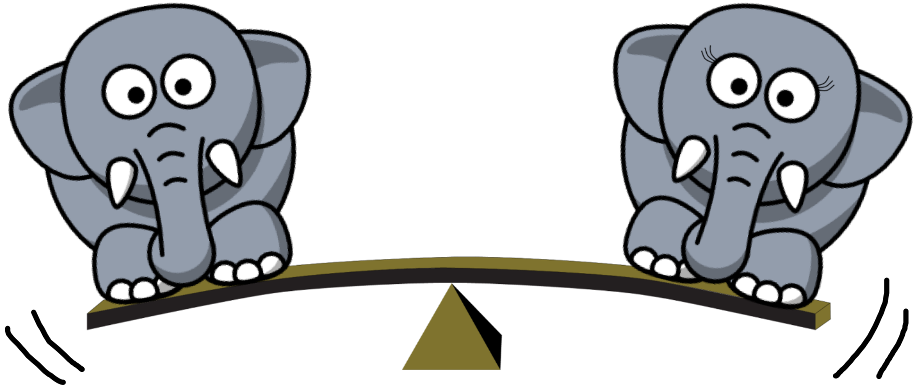

Normal Reactive Scope decreases.
= wear-and-tear

Reduced tolerance for additional physiological pressure.

The body does not normally respond in the *Homeostatic Overload* range, unless…

Reactive Scope Model

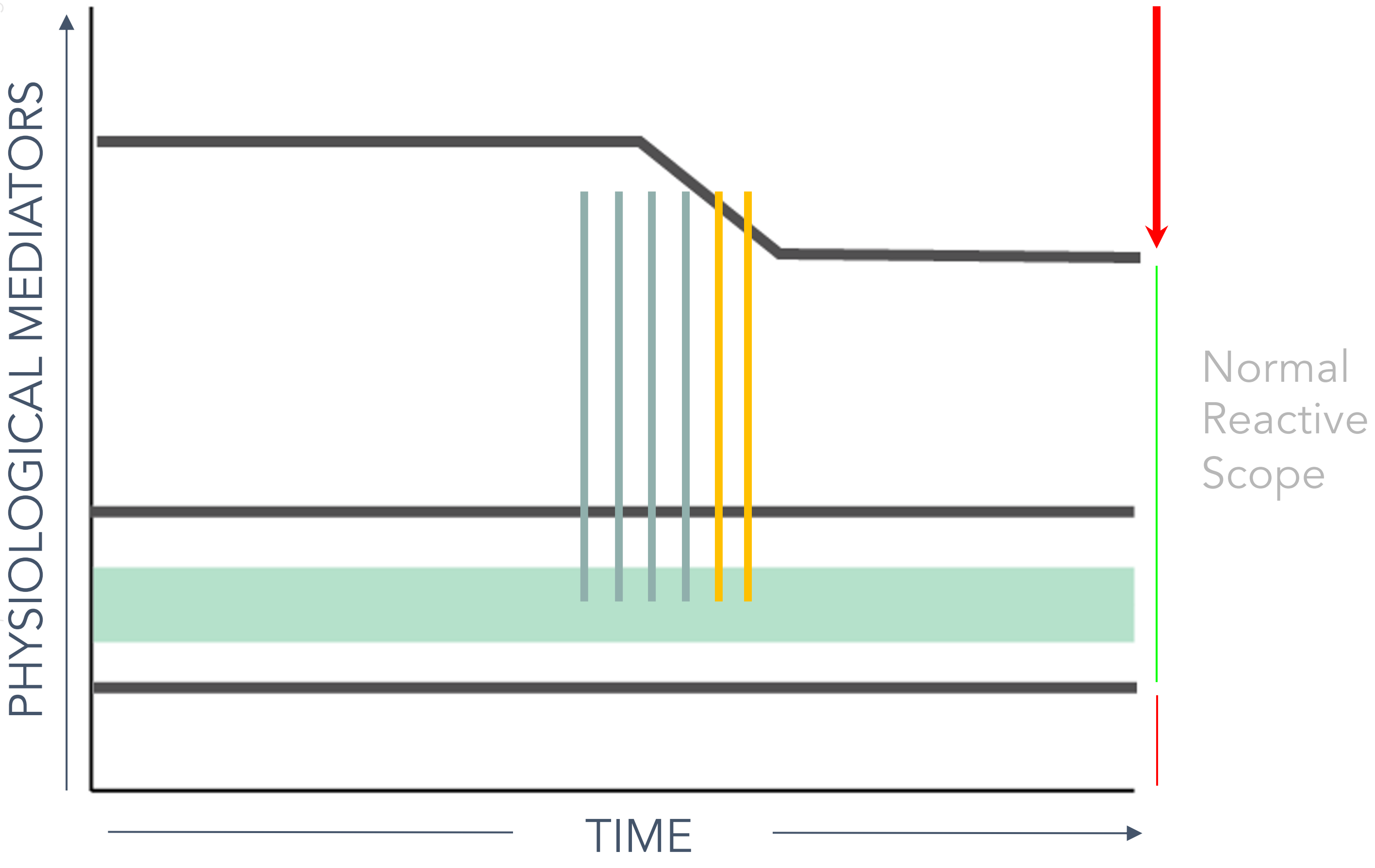

From: Dickens MJ, Pawluski JL and Romero LM (2020) Moving Forward From COVID-19: Bridging Knowledge Gaps in Maternal Health With a New Conceptual Model. Front. Glob. Womens Health 1:586697. doi: 10.3389/fgwh.2020.586697

…the physiological mediators are pushed ***beyond normal functionality*** and overcompensate to maintain balance.

This can occur due to:

A series of acute stress responses

Mediators don't have capacity to fully recover between responses.

The body does not normally respond in the *Homeostatic Overload* range, unless...

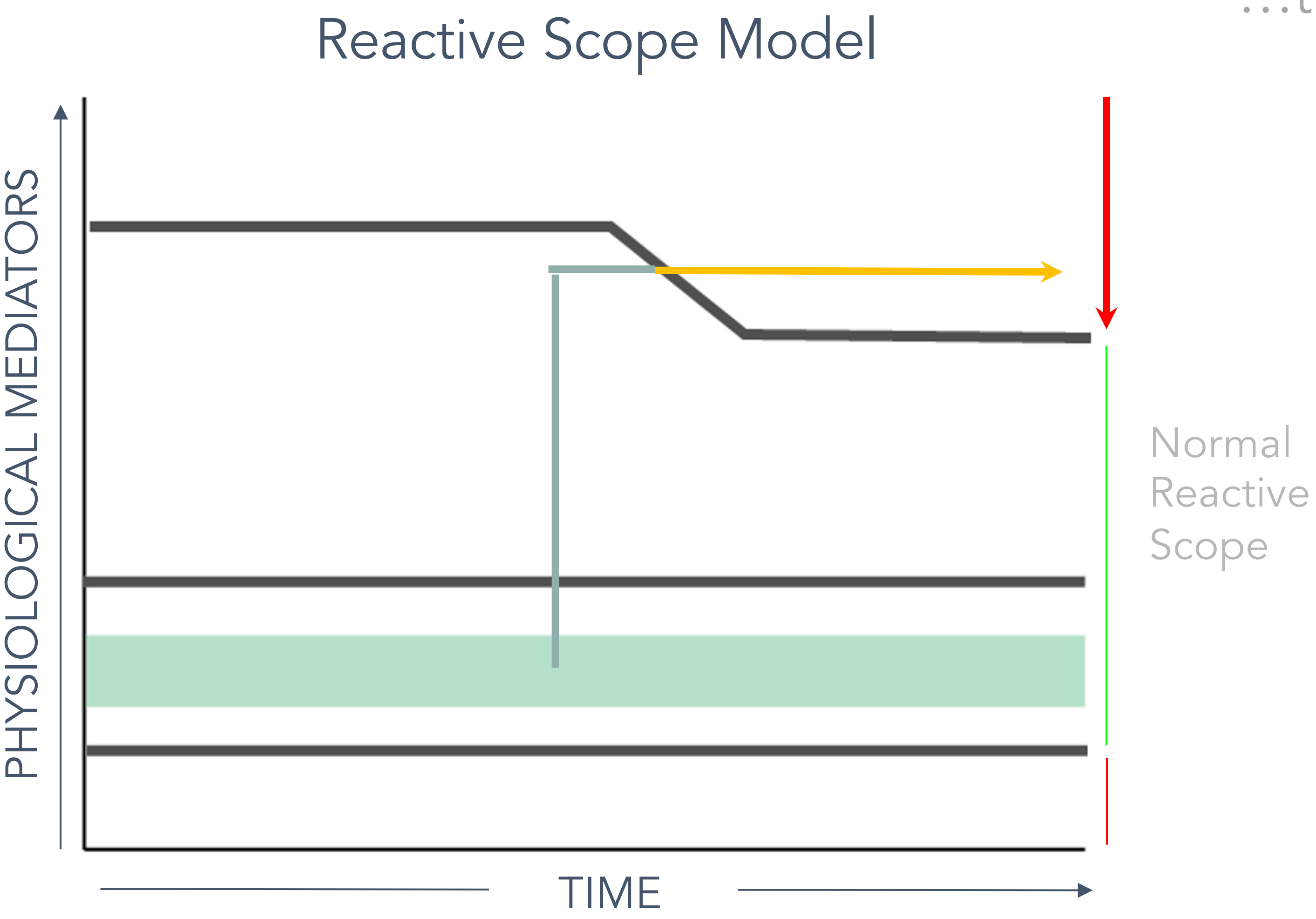

...the physiological mediators are pushed *beyond normal functionality* and overcompensate to maintain balance.

OR:

Chronic activation of the stress response

Mediators are continuously operating at elevated levels.

From: Dickens MJ, Pawluski JL and Romero LM (2020) Moving Forward From COVID-19: Bridging Knowledge Gaps in Maternal Health With a New Conceptual Model. Front. Glob. Womens Health 1:586697. doi: 10.3389/fgwh.2020.586697

The body does not normally respond in the *Homeostatic Overload* range, unless…

Reactive Scope Model

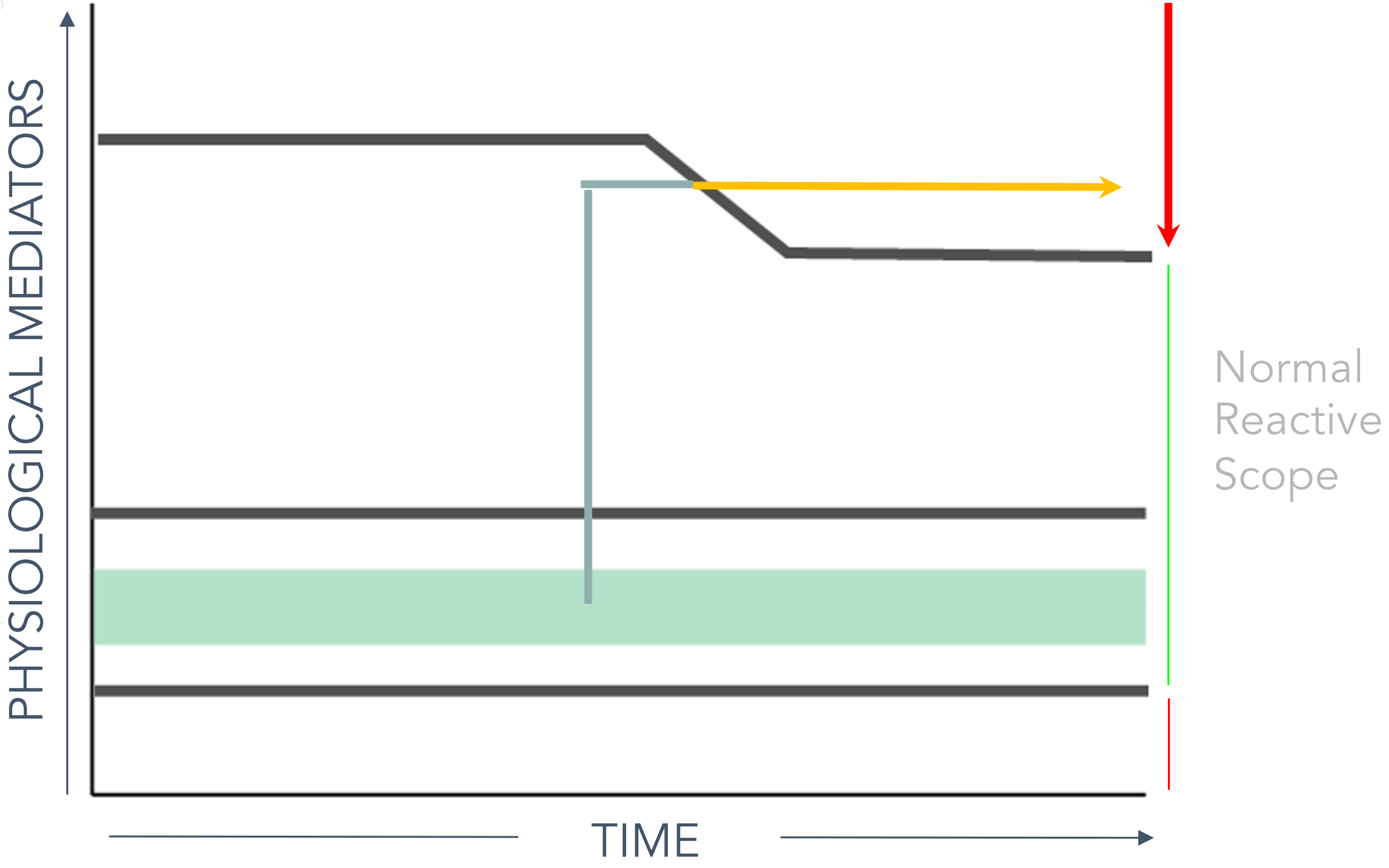

From: Dickens MJ, Pawluski JL and Romero LM (2020) Moving Forward From COVID-19: Bridging Knowledge Gaps in Maternal Health With a New Conceptual Model. Front. Glob. Womens Health 1:586697. doi: 10.3389/fgwh.2020.586697

…the physiological mediators are pushed *beyond normal functionality* and overcompensate to maintain balance.

This state has also been called:

Chronic Stress
Allostatic Overload

Or, often, simply,

STRESS.

= body prone to stress-related illness

Pregnancy makes homeostasis even more complicated....

From: *Dickens MJ, Pawluski JL and Romero LM (2020) Moving Forward From COVID-19: Bridging Knowledge Gaps in Maternal Health With a New Conceptual Model. Front. Glob. Womens Health 1:586697. doi: 10.3389/fgwh.2020.586697*

It’s nice to think in straight lines, BUT, most systems don’t function this way…

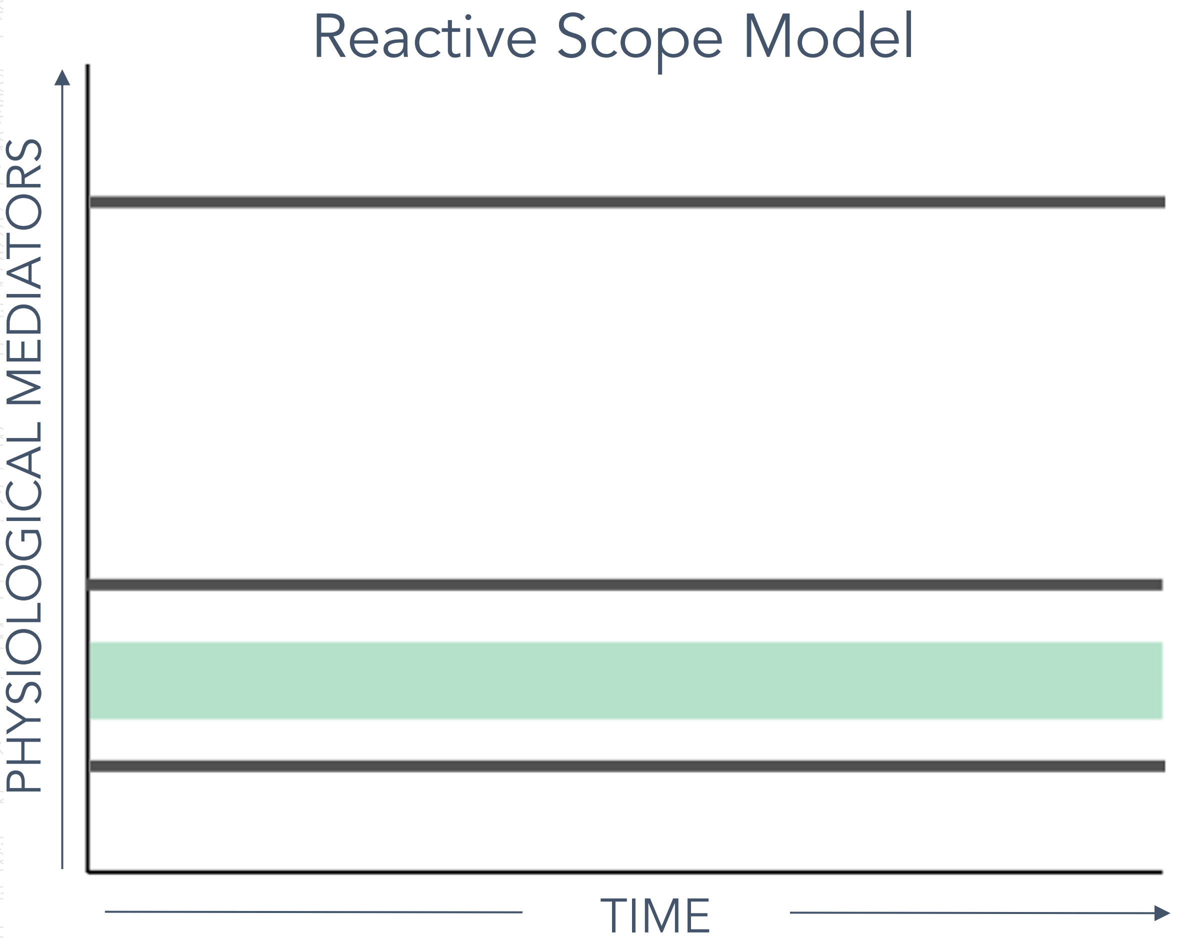

From: Dickens MJ, Pawluski JL and Romero LM (2020) Moving Forward From COVID-19: Bridging Knowledge Gaps in Maternal [illegible] 2020.586697

It's nice to think in straight lines, BUT, most systems don't function this way…

…like during pregnancy*.

Reactive Scope Model

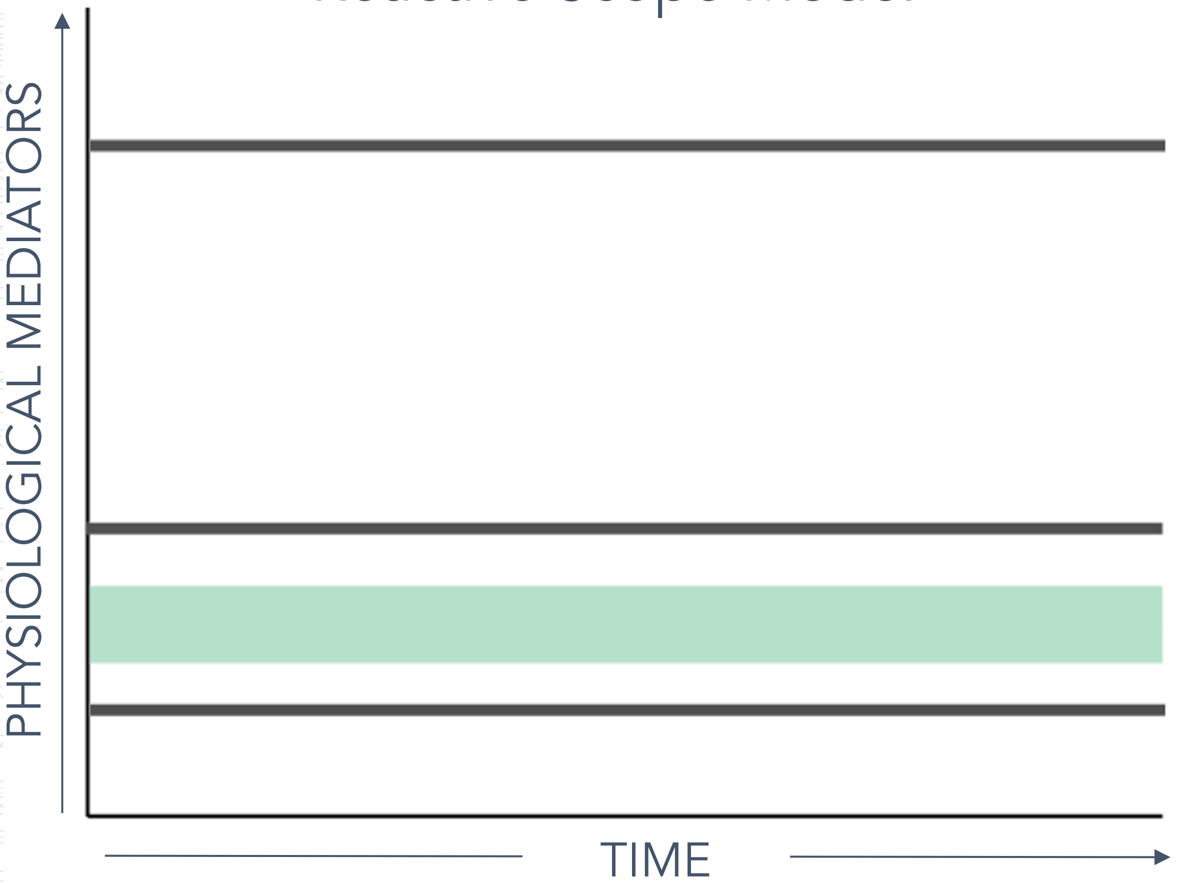

Maternal Reactive Scope Model

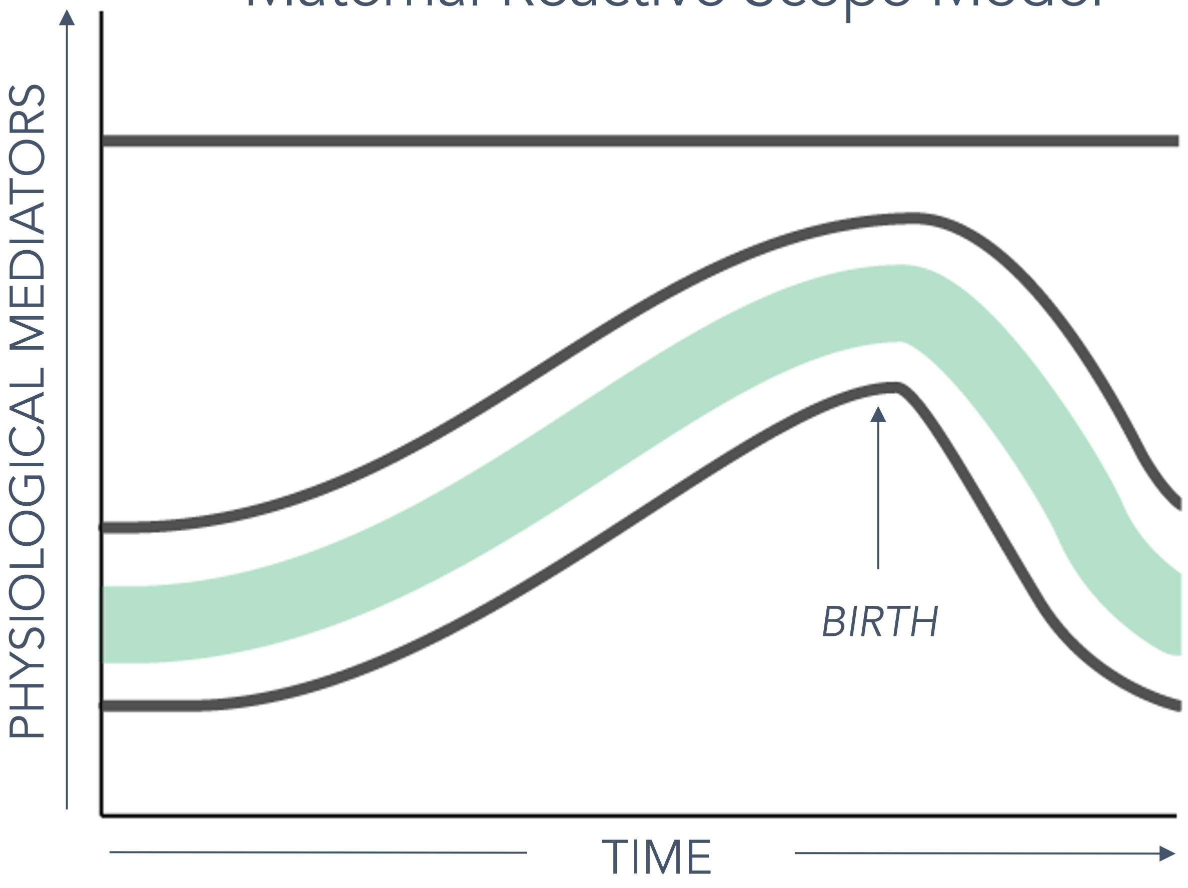

*Note: with significant gaps in the literature on pregnancy physiology, we are generalizing the patterns of mediator levels. We see this model as a *starting point* for future work and adaptation. See the appendix for examples of physiological mediators and potential directionality

From: Dickens MJ, Pawluski JL and Romero LM (2020) Moving Forward From COVID-19: Bridging Knowledge Gaps in Maternal [illegible] 2020.586697

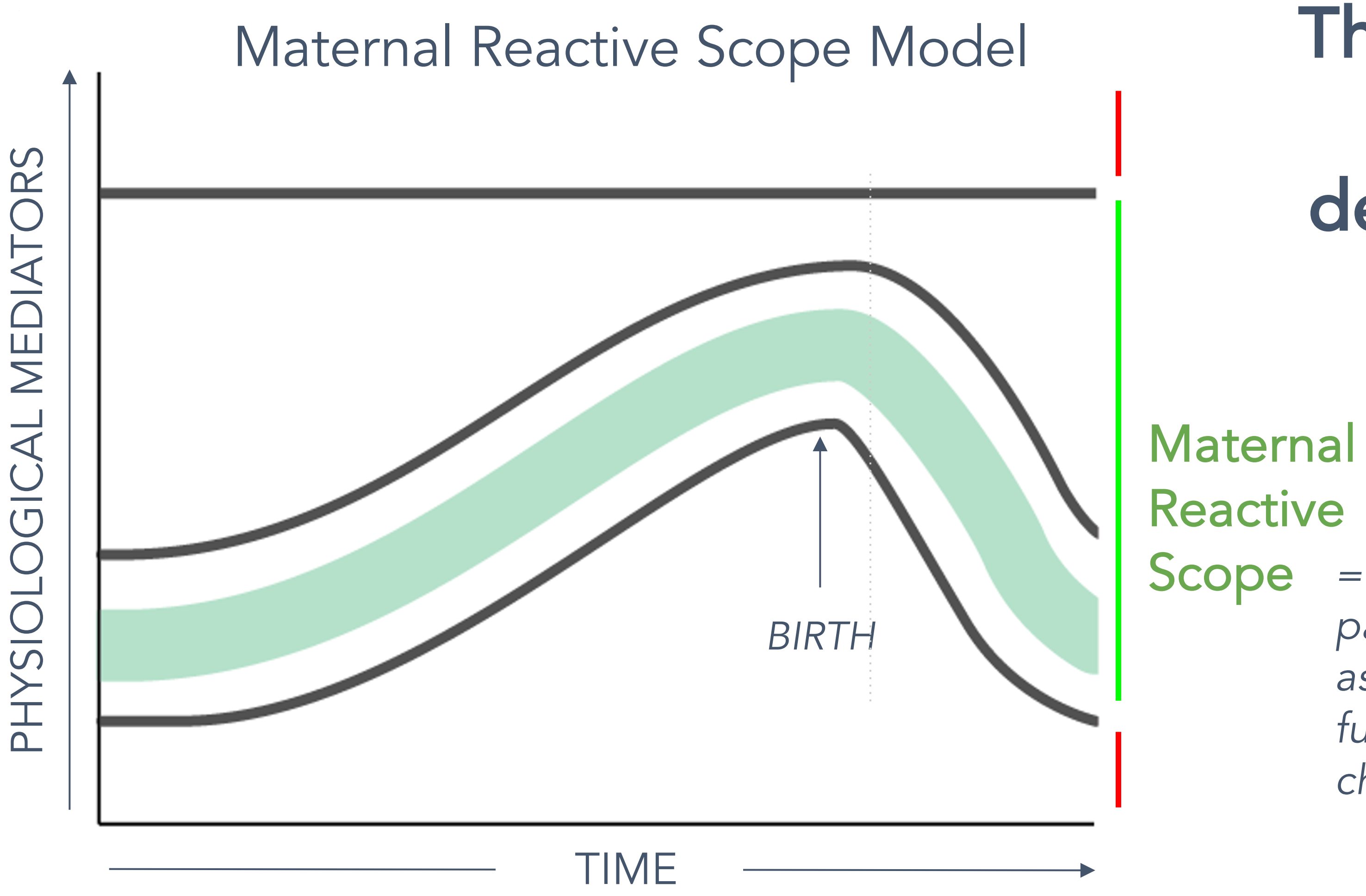

= natural changes in physiological parameters across pregnancy are reflected as increasing requirements to maintain daily function and respond to predictable challenges

The maternal body shifts to prioritize the growth, development, delivery, and feeding of a new human.

From: Dickens MJ, Pawluski JL and Romero LM (2020) Moving Forward From COVID-19: Bridging Knowledge Gaps in Maternal Health With a New Conceptual Model. Front. Glob. Womens Health 1:586697. doi: 10.3389/fgwh.2020.586697

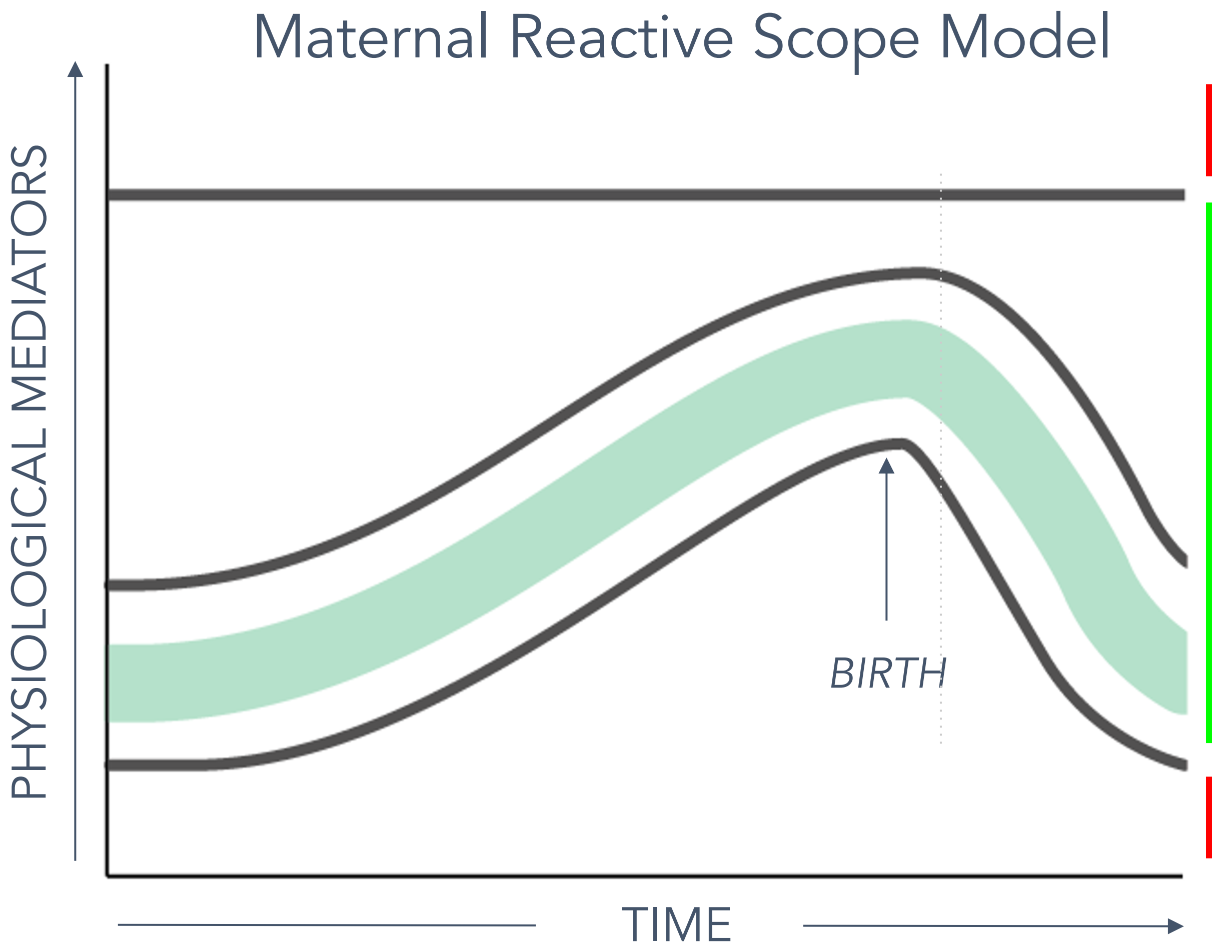

From: Dickens MJ, Pawluski JL and Romero LM (2020) Moving Forward From COVID-19: Bridging Knowledge Gaps in Maternal Health With a New Conceptual Model. Front. Glob. Womens Health 1:586697. doi: 10.3389/fgwh.2020.586697

The maternal body shifts to prioritize the growth, development, delivery, and feeding of a new human.

Predictive Homeostasis range shift = required to maintain healthy pregnancy, prepare for labor and delivery, and support postpartum needs.

Pregnancy is not a disease, but...

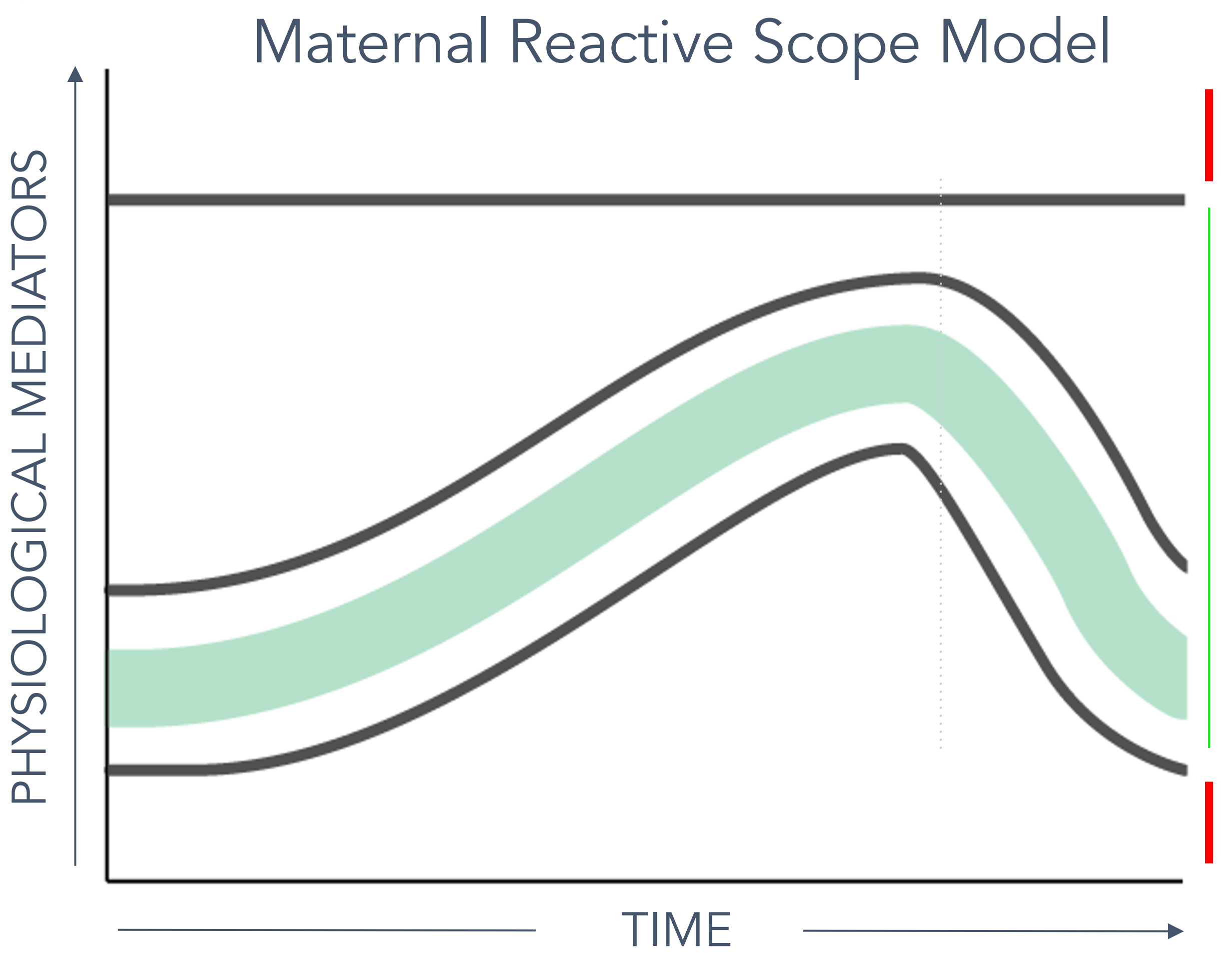

From: Dickens MJ, Pawluski JL and Romero LM (2020) Moving Forward From COVID-19: Bridging Knowledge Gaps in Maternal Health With a New Conceptual Model. Front. Glob. Womens Health 1:586697. doi: 10.3389/fgwh.2020.586697

...it makes the body more ***vulnerable*** to pathology.

Inability to maintain adequate mediator levels can lead to **Homeostatic Failure**

Stress responses more easily cross into **Homeostatic Overload**

Pregnancy is not a disease, but it makes the body more ***vulnerable*** to pathology.

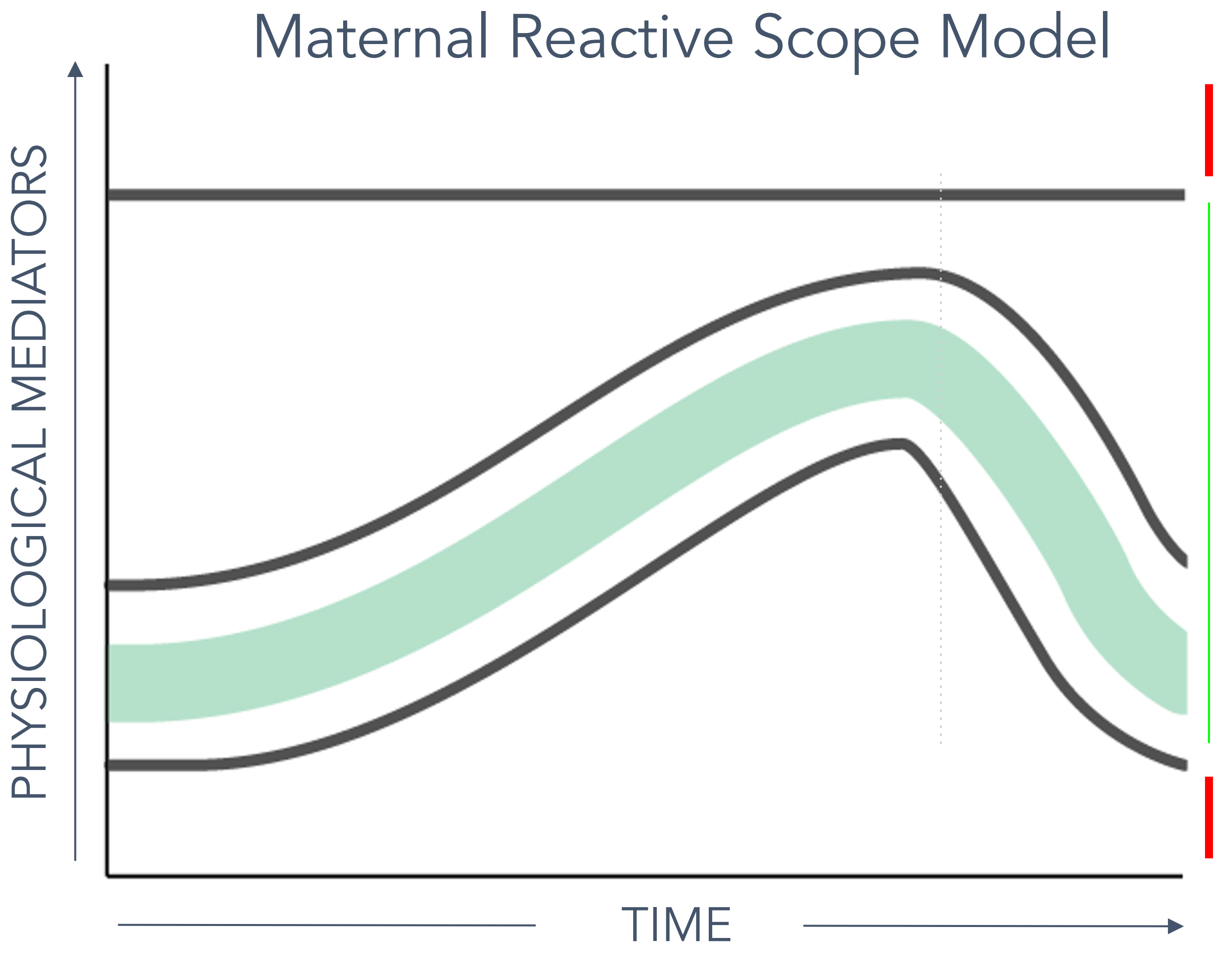

From: Dickens MJ, Pawluski JL and Romero LM (2020) Moving Forward From COVID-19: Bridging Knowledge Gaps in Maternal Health With a New Conceptual Model. Front. Glob. Womens Health 1:586697. doi: 10.3389/fgwh.2020.586697

Homeostatic Failure/Overload will present as illness, pregnancy complications, and/or developmental issues

For example:*

- Gestational Diabetes
- Hypertension, preeclampsia
- Preterm birth
- Anxiety/depression
- Cardiovascular disease

*See the appendix for examples of physiological mediators and changes during perinatal period which may link to health complications.

Pregnancy is not a disease, but it makes the body more *vulnerable* to pathology.

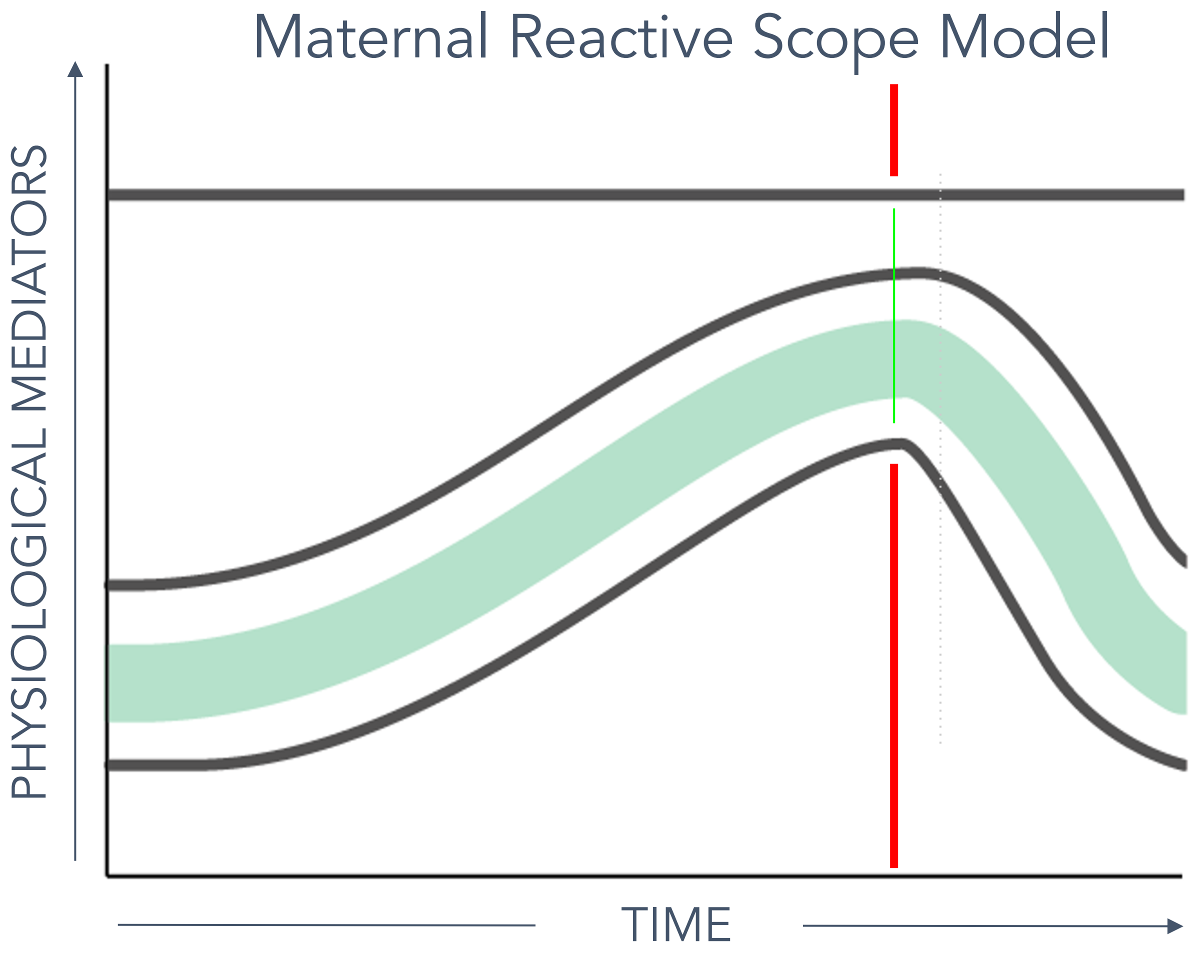

Highest risk towards the end of pregnancy into early postpartum when the ***Maternal Reactive Scope*** is narrowest

- Physiological requirements to sustain the health of mom, baby, and the maternal<>fetal unit *and* maintain homeostatic balance becomes more precarious as the maternal reactive scope is naturally compressed

From: Dickens MJ, Pawluski JL and Romero LM (2020) Moving Forward From COVID-19: Bridging Knowledge Gaps in Maternal Health With a New Conceptual Model. Front. Glob. Womens Health 1:586697. doi: 10.3389/fgwh.2020.586697

Stress and pregnancy

From: Dickens MJ, Pawluski JL and Romero LM (2020) Moving Forward From COVID-19: Bridging Knowledge Gaps in Maternal Health With a New Conceptual Model. Front. Glob. Womens Health 1:586697. doi: 10.3389/fgwh.2020.586697

Acute stress during pregnancy

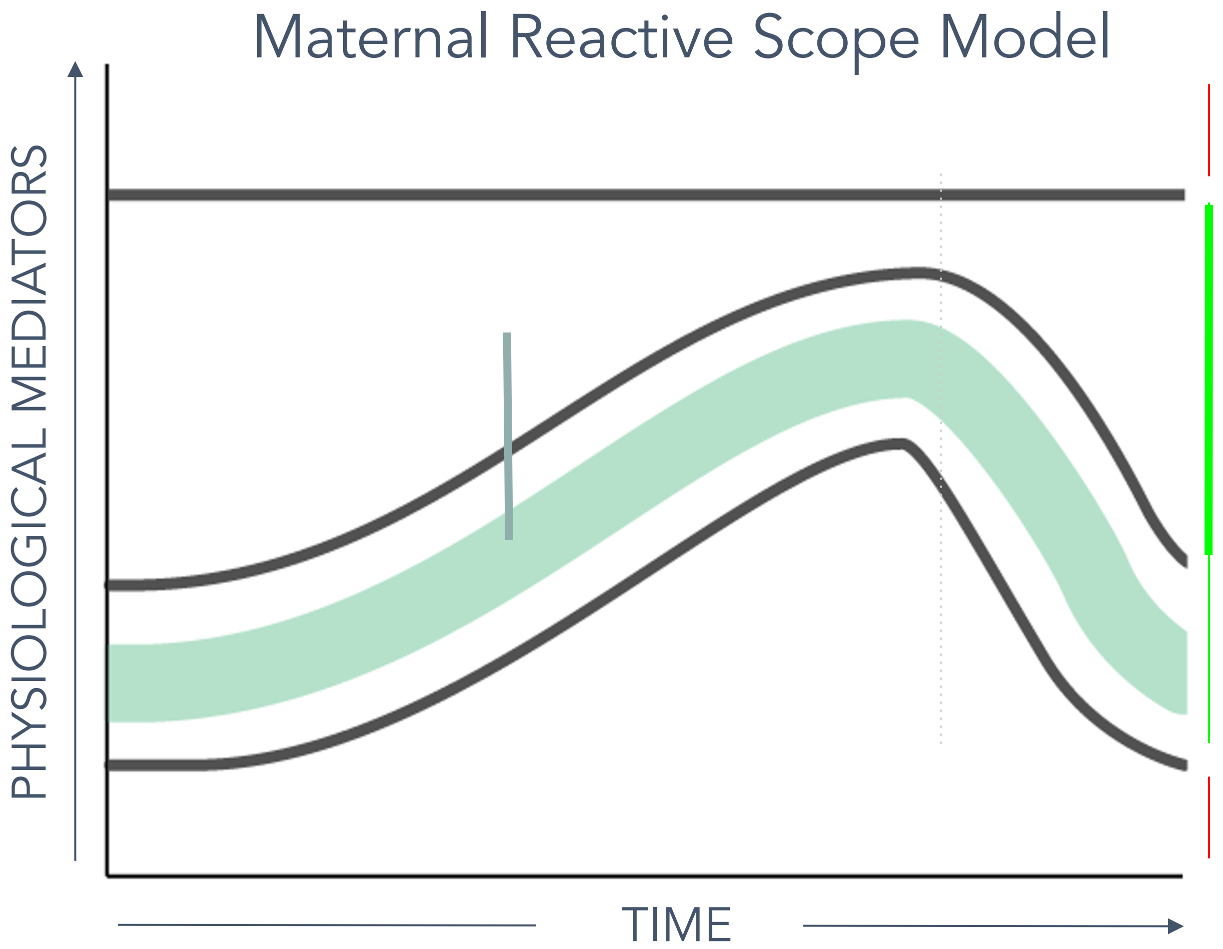

From: Dickens MJ, Pawluski JL and Romero LM (2020) Moving Forward From COVID-19: Bridging Knowledge Gaps in Maternal Health With a New Conceptual Model. Front. Glob. Womens Health 1:586697. doi: 10.3389/fgwh.2020.586697

A healthy acute stress response typically operates in the *Reactive Homeostasis* range.

Acute stress during pregnancy

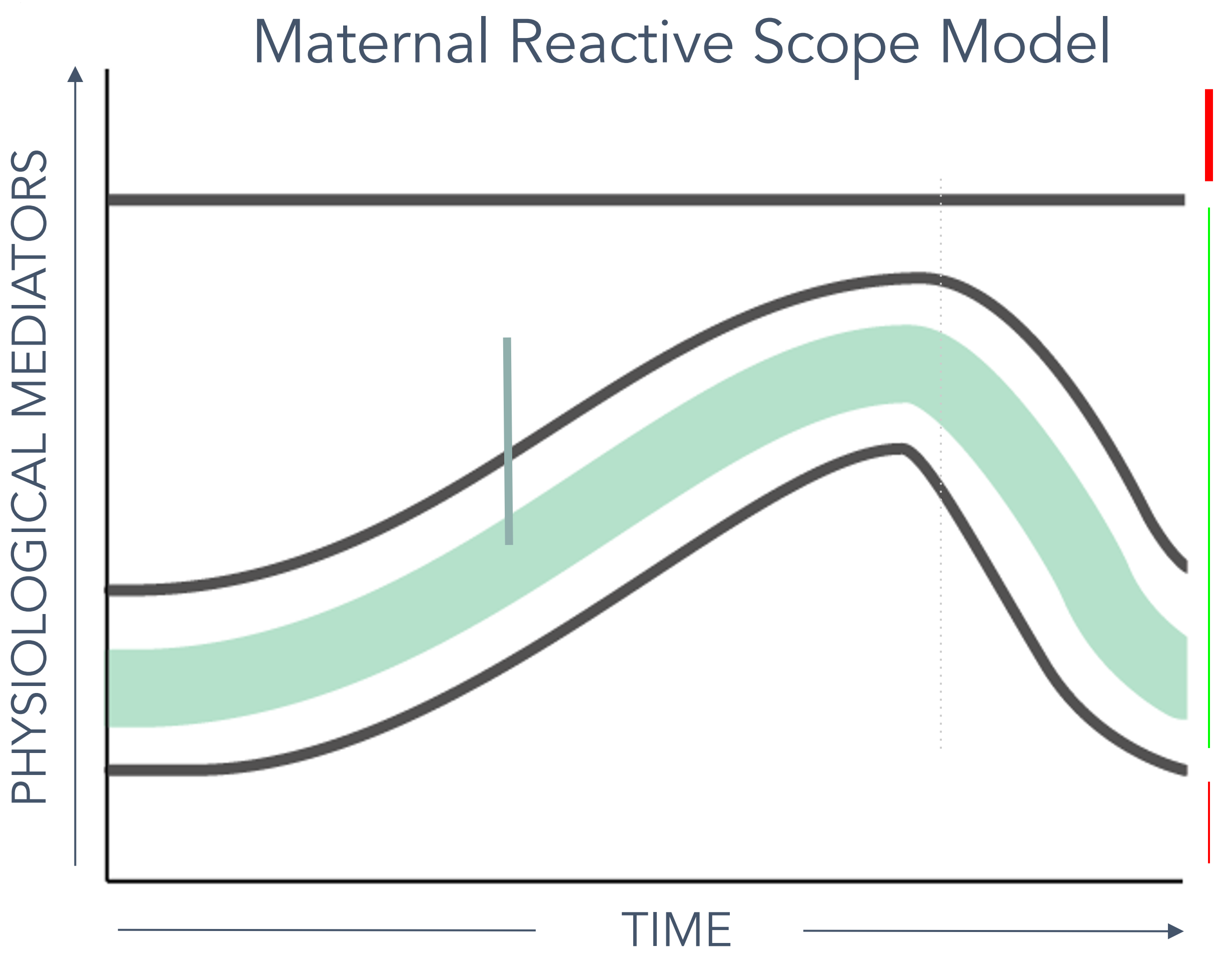

From: Dickens MJ, Pawluski JL and Romero LM (2020) Moving Forward From COVID-19: Bridging Knowledge Gaps in Maternal Health With a New Conceptual Model. Front. Glob. Womens Health 1:586697. doi: 10.3389/fgwh.2020.586697

But an acute stress response can enter, *Homeostatic Overload* in response to:

Acute stress during pregnancy

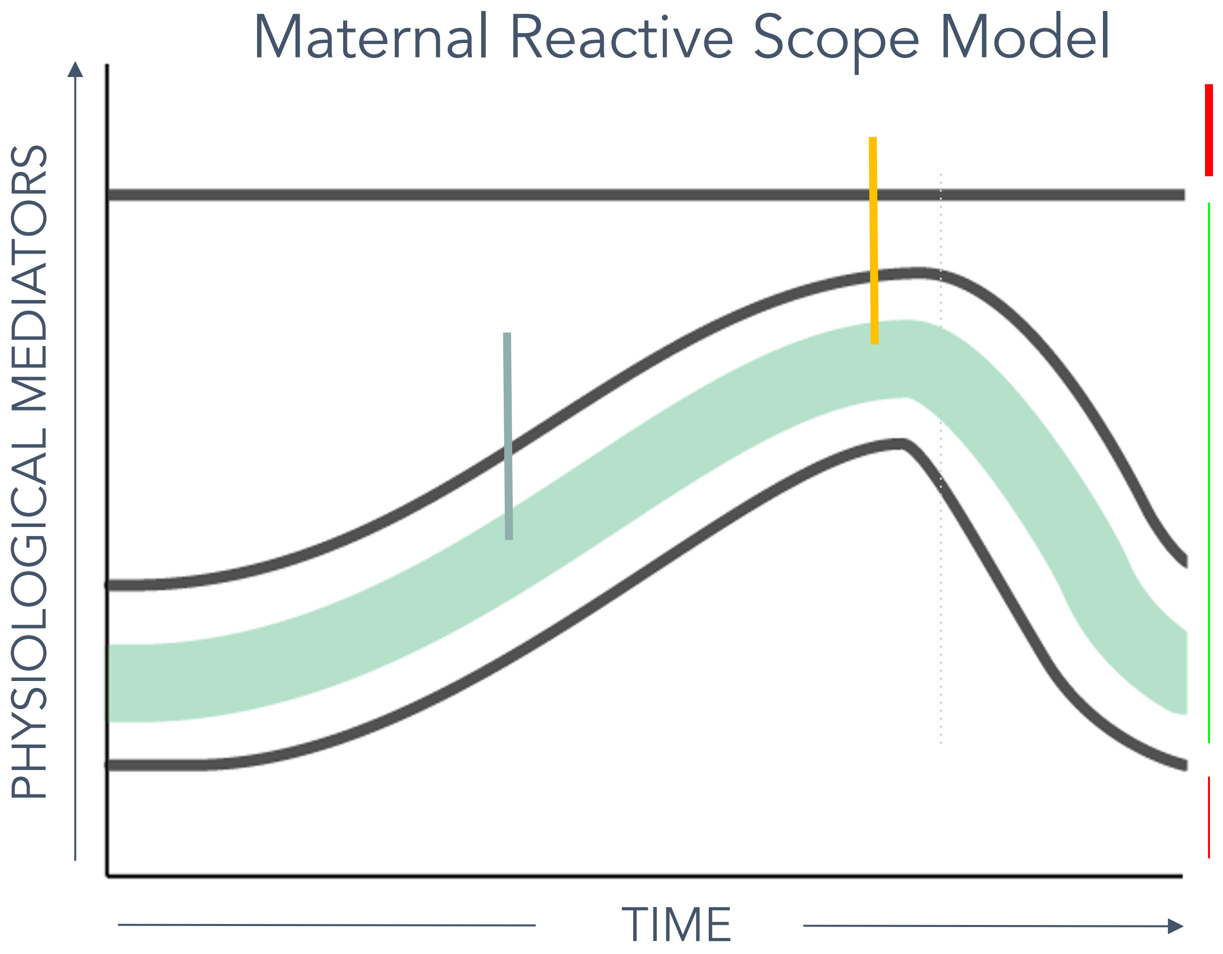

From: Dickens MJ, Pawluski JL and Romero LM (2020) Moving Forward From COVID-19: Bridging Knowledge Gaps in Maternal Health With a New Conceptual Model. Front. Glob. Womens Health 1:586697. doi: 10.3389/fgwh.2020.586697

But an acute stress response can enter, *Homeostatic Overload* in response to:

- **Stress exposure when the *maternal reactive scope* is compressed**

Acute stress during pregnancy

Maternal Reactive Scope Model

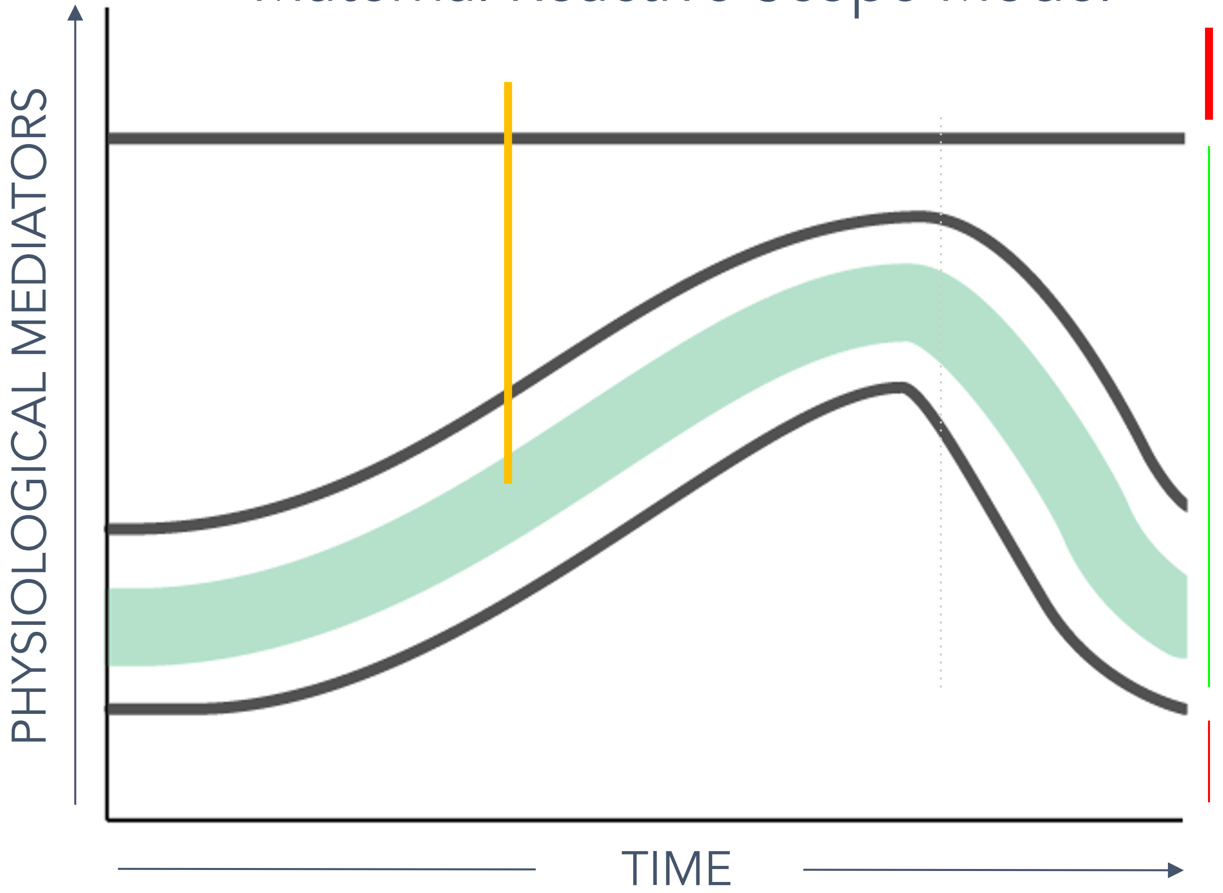

From: Dickens MJ, Pawluski JL and Romero LM (2020) Moving Forward From COVID-19: Bridging Knowledge Gaps in Maternal Health With a New Conceptual Model. Front. Glob. Womens Health 1:586697. doi: 10.3389/fgwh.2020.586697

But an acute stress response can enter, *Homeostatic Overload* in response to:

- Stress exposure when the *maternal reactive scope* is compressed
- **A single strong stress event (e.g. trauma)**

Chronic stress during pregnancy

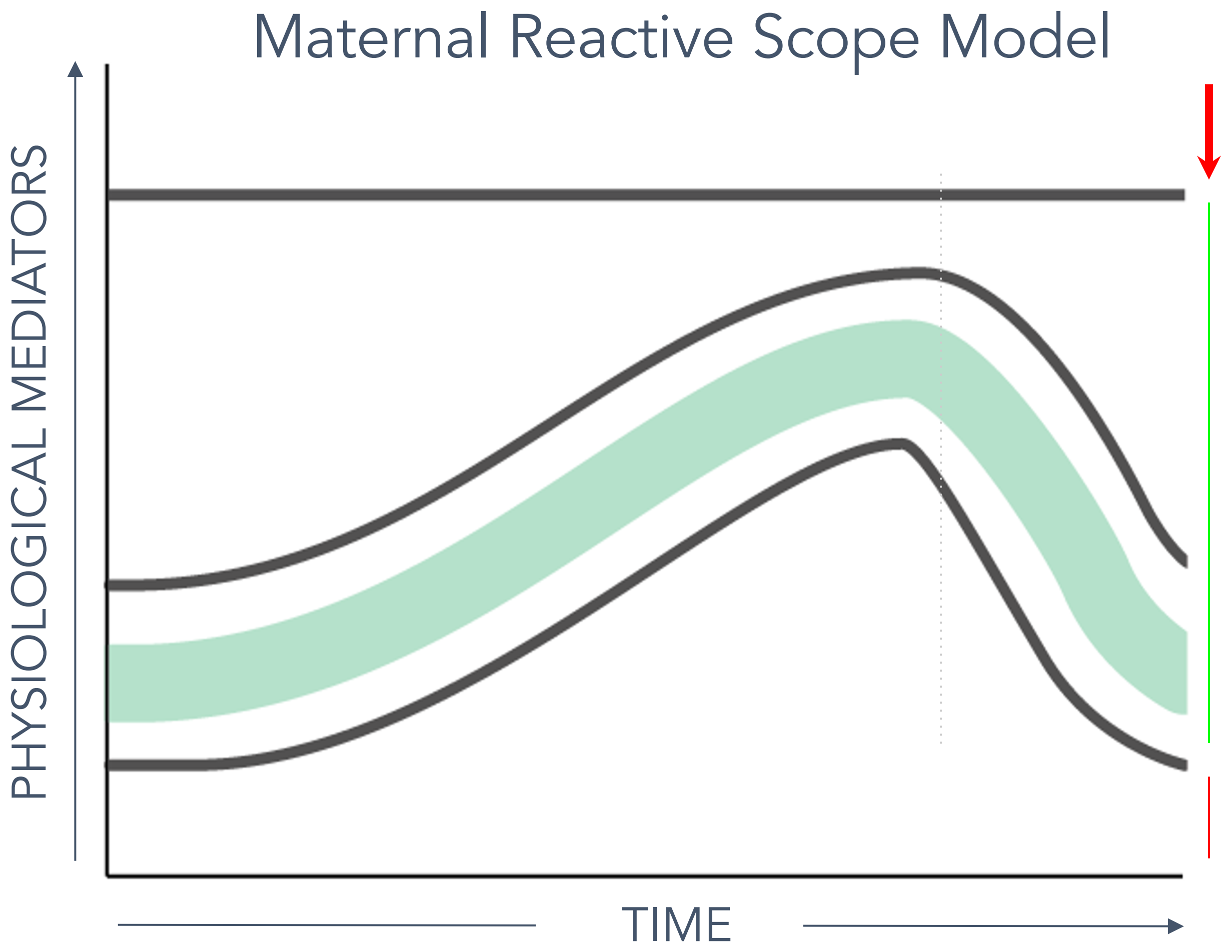

From: Dickens MJ, Pawluski JL and Romero LM (2020) Moving Forward From COVID-19: Bridging Knowledge Gaps in Maternal Health With a New Conceptual Model. Front. Glob. Womens Health 1:586697. doi: 10.3389/fgwh.2020.586697

Homeostatic Overload also occurs when the upper threshold shifts *down* in response to:

Chronic stress during pregnancy

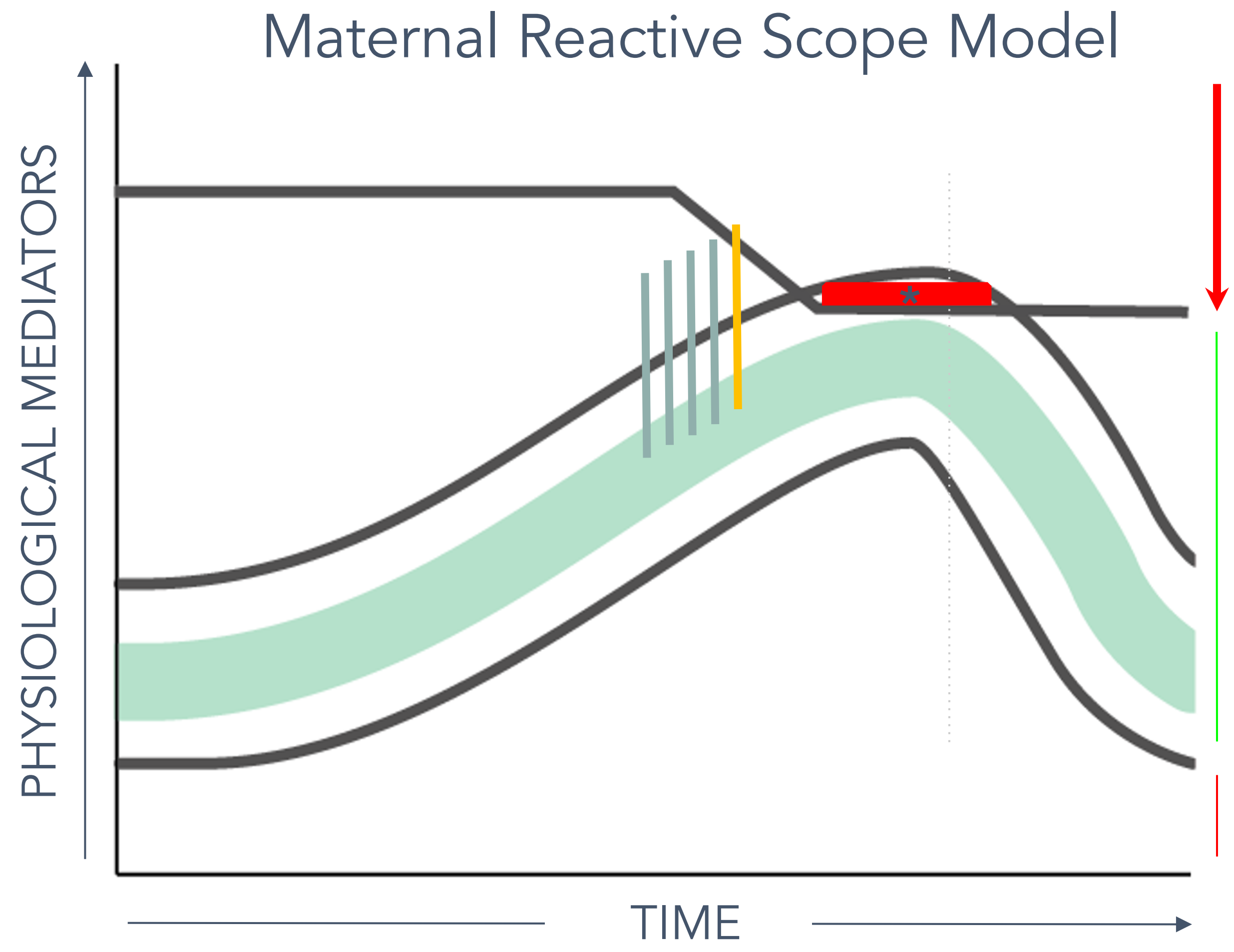

From: Dickens MJ, Pawluski JL and Romero LM (2020) Moving Forward From COVID-19: Bridging Knowledge Gaps in Maternal Health With a New Conceptual Model. Front. Glob. Womens Health 1:586697. doi: 10.3389/fgwh.2020.586697

Homeostatic Overload **also occurs when the upper threshold shifts *down* in response to:**

- **A series of stressors without recovery**

*Depending on the degree to which the *maternal reactive scope* is compressed, normal functionality of mediators operating in the Predictive Homeostasis range may affect health.

Chronic stress during pregnancy

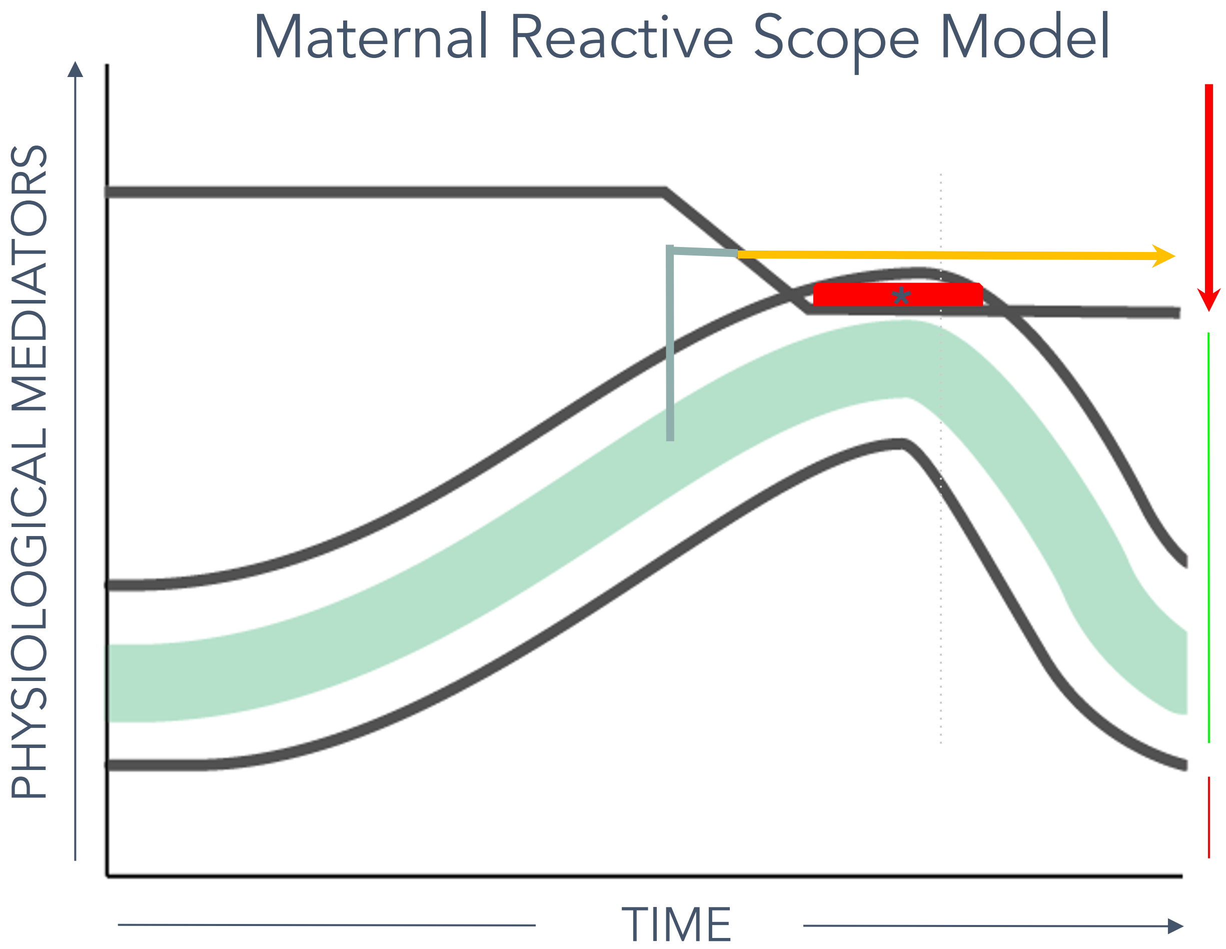

From: Dickens MJ, Pawluski JL and Romero LM (2020) Moving Forward From COVID-19: Bridging Knowledge Gaps in Maternal Health With a New Conceptual Model. Front. Glob. Womens Health 1:586697. doi: 10.3389/fgwh.2020.586697

Homeostatic Overload
also occurs when the upper threshold shifts *down* in response to:

- A series of stressors without recovery
- **Continuous activation**

*Depending on the degree to which the *maternal reactive scope* is compressed, normal functionality of mediators operating in the Predictive Homeostasis range may affect health.

Chronic stress during pregnancy

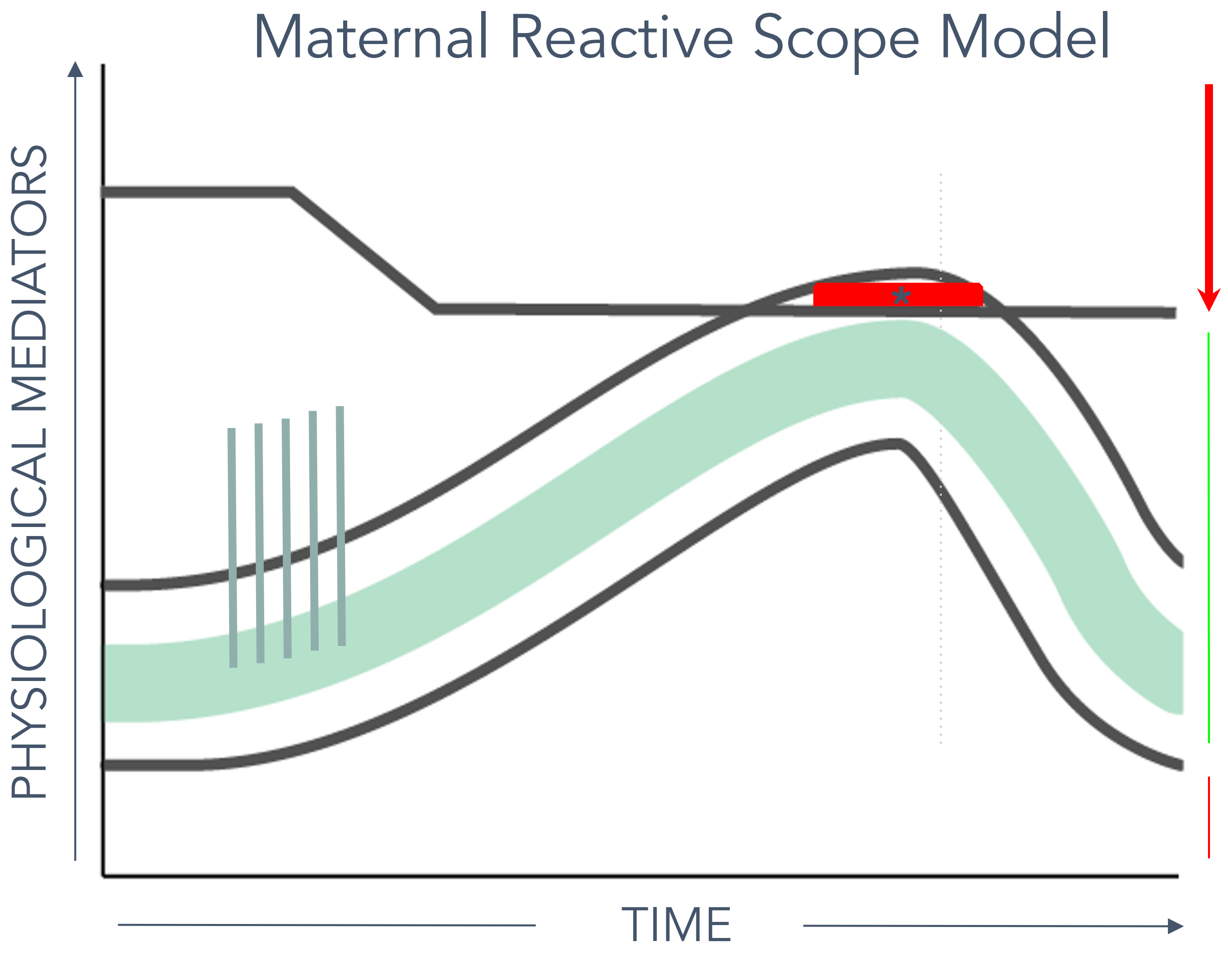

From: Dickens MJ, Pawluski JL and Romero LM (2020) Moving Forward From COVID-19: Bridging Knowledge Gaps in Maternal Health With a New Conceptual Model. Front. Glob. Womens Health 1:586697. doi: 10.3389/fgwh.2020.586697

Homeostatic Overload **also occurs when the upper threshold shifts *down* in response to:**

- A series of stressors without recovery
- Continuous activation
- **Early pregnancy-related stress with long-lasting effects**

*Depending on the degree to which the *maternal reactive scope* is compressed, normal functionality of mediators operating in the Predictive Homeostasis range may affect health.

An individual's upper threshold may be lower *prior to pregnancy*...

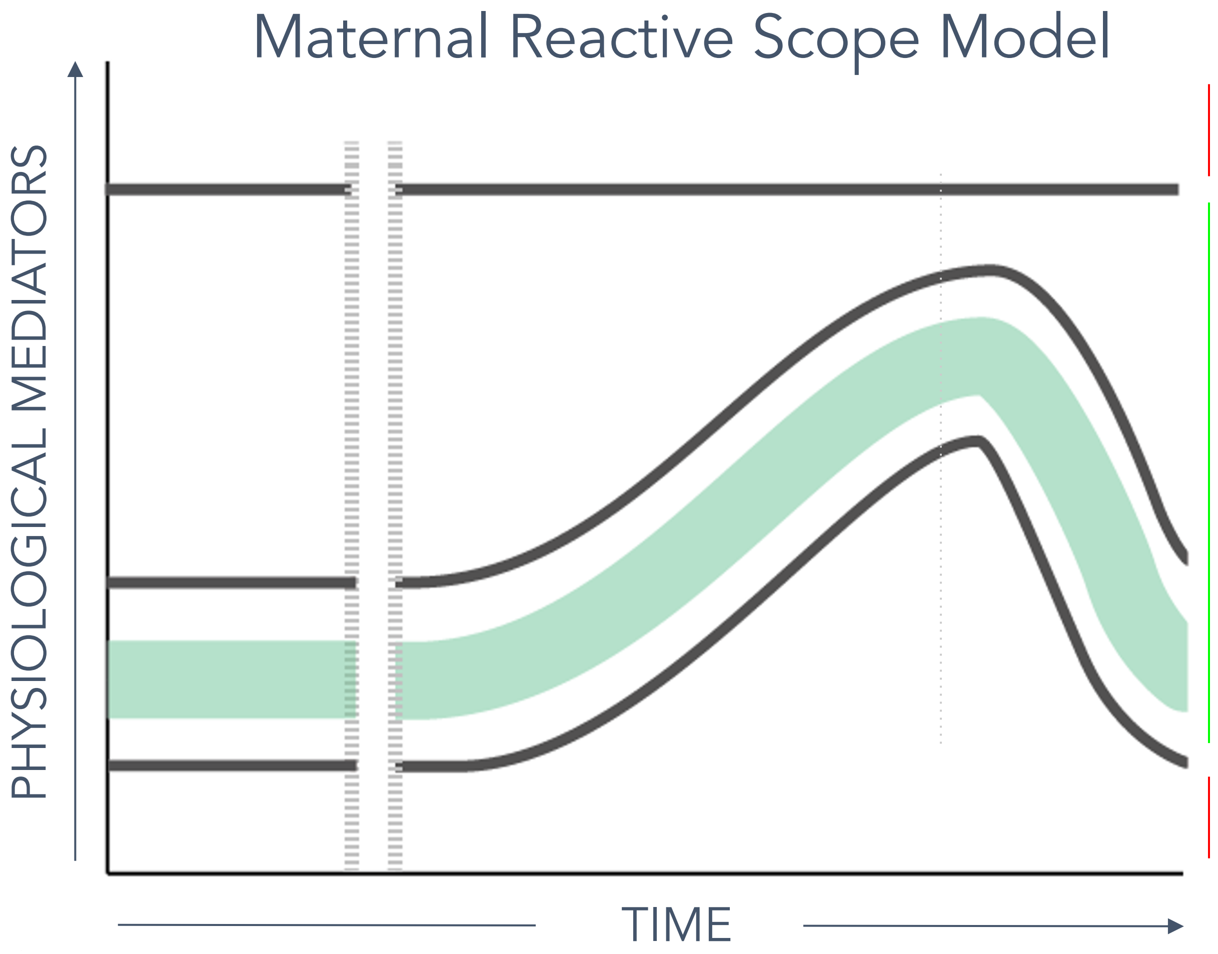

From: Dickens MJ, Pawluski JL and Romero LM (2020) Moving Forward From COVID-19: Bridging Knowledge Gaps in Maternal Health With a New Conceptual Model. Front. Glob. Womens Health 1:586697. doi: 10.3389/fgwh.2020.586697

Upper threshold may be lower *prior to pregnancy*...

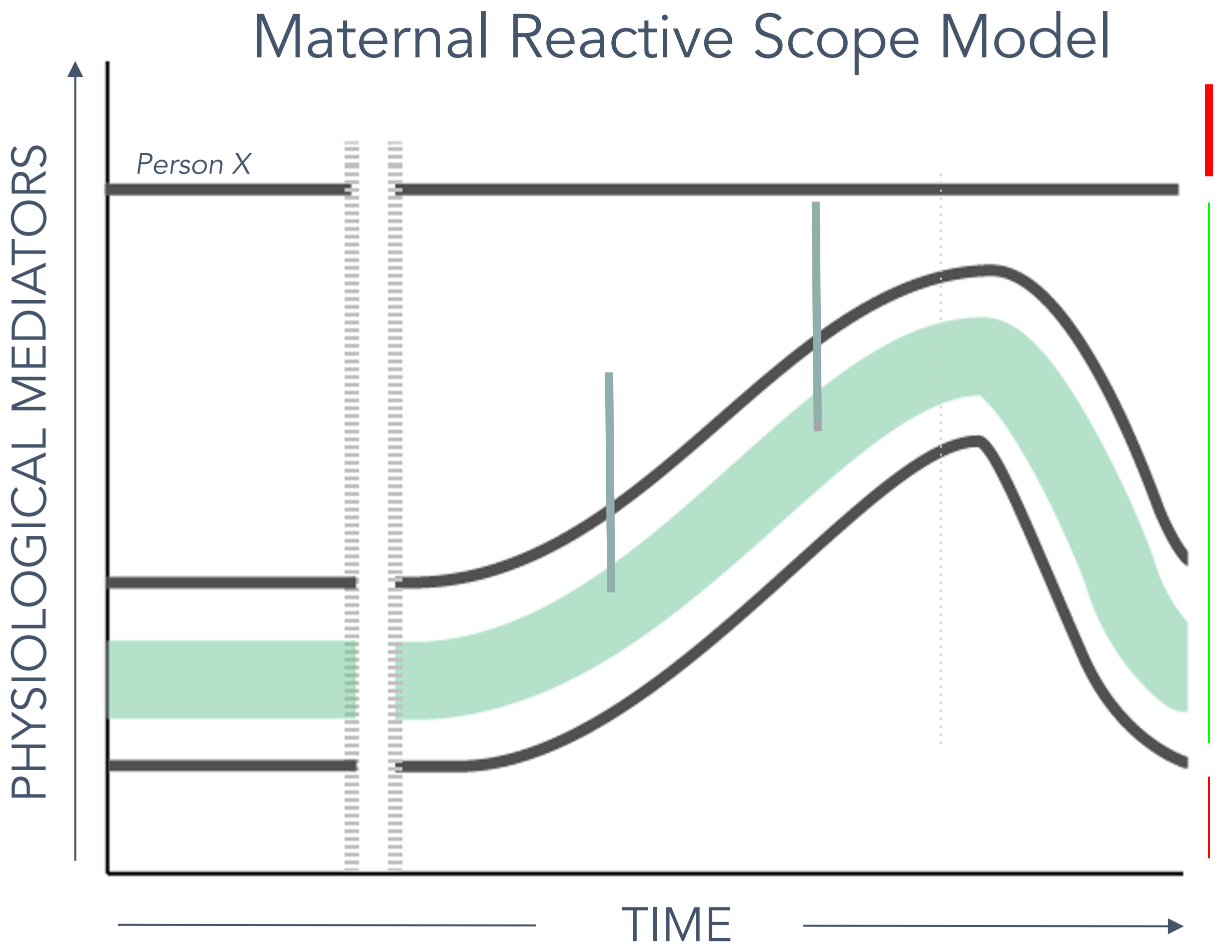

From: Dickens MJ, Pawluski JL and Romero LM (2020) Moving Forward From COVID-19: Bridging Knowledge Gaps in Maternal Health With a New Conceptual Model. Front. Glob. Womens Health 1:586697. doi: 10.3389/fgwh.2020.586697

Individual differences may increase risk of *Homeostatic Overload:*

Upper threshold may be lower *prior to pregnancy…*

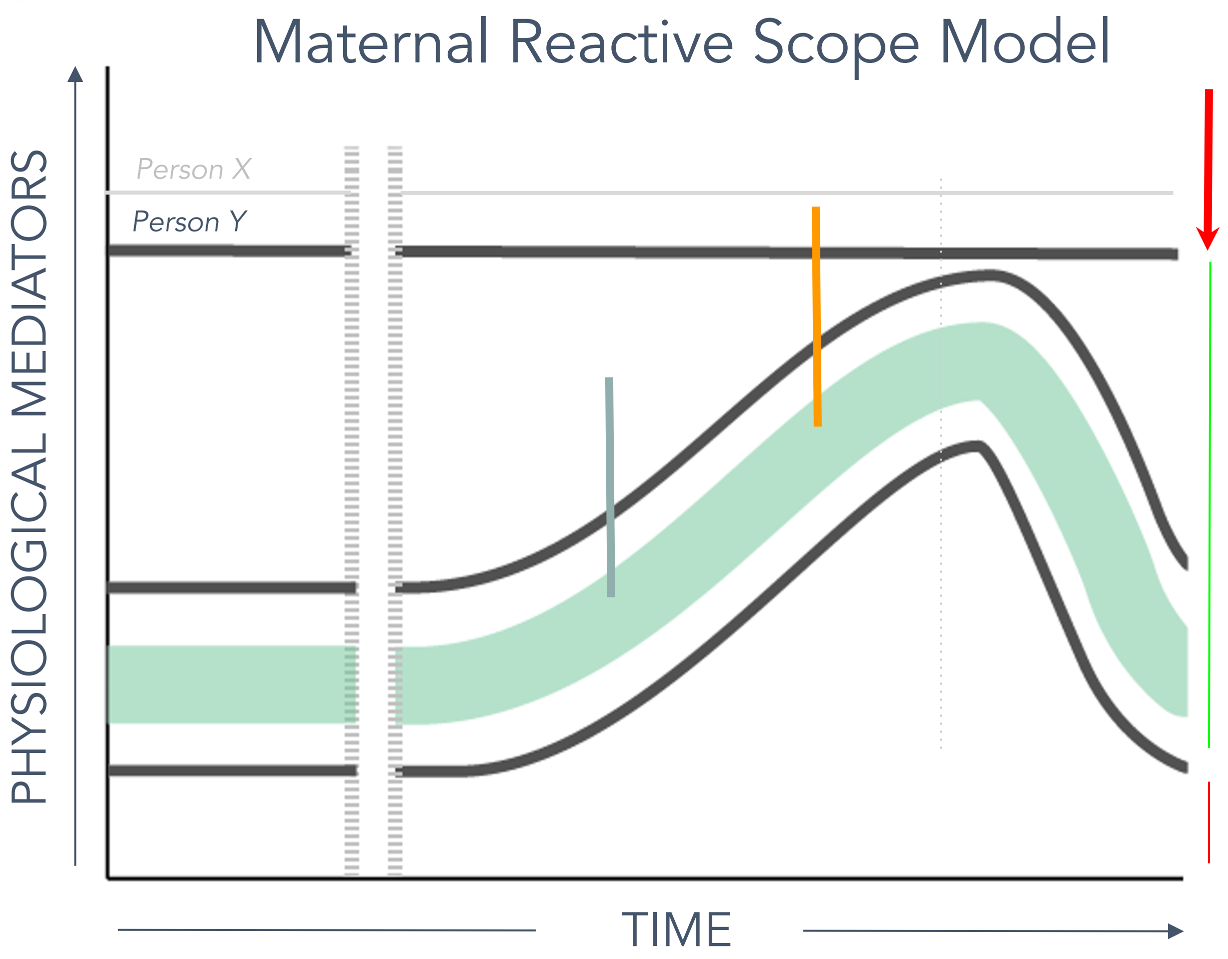

From: Dickens MJ, Pawluski JL and Romero LM (2020) Moving Forward From COVID-19: Bridging Knowledge Gaps in Maternal Health With a New Conceptual Model. Front. Glob. Womens Health 1:586697. doi: 10.3389/fgwh.2020.586697

Individual differences may increase risk of *Homeostatic Overload:*

- **Genetic susceptibility**

Certain individuals may be ***more sensitive or more resilient to stressors*** *during pregnancy*

(e.g. environmental stressors could increases risk of perinatal depression/anxiety for some women)

Upper threshold may be lower *prior to pregnancy...*

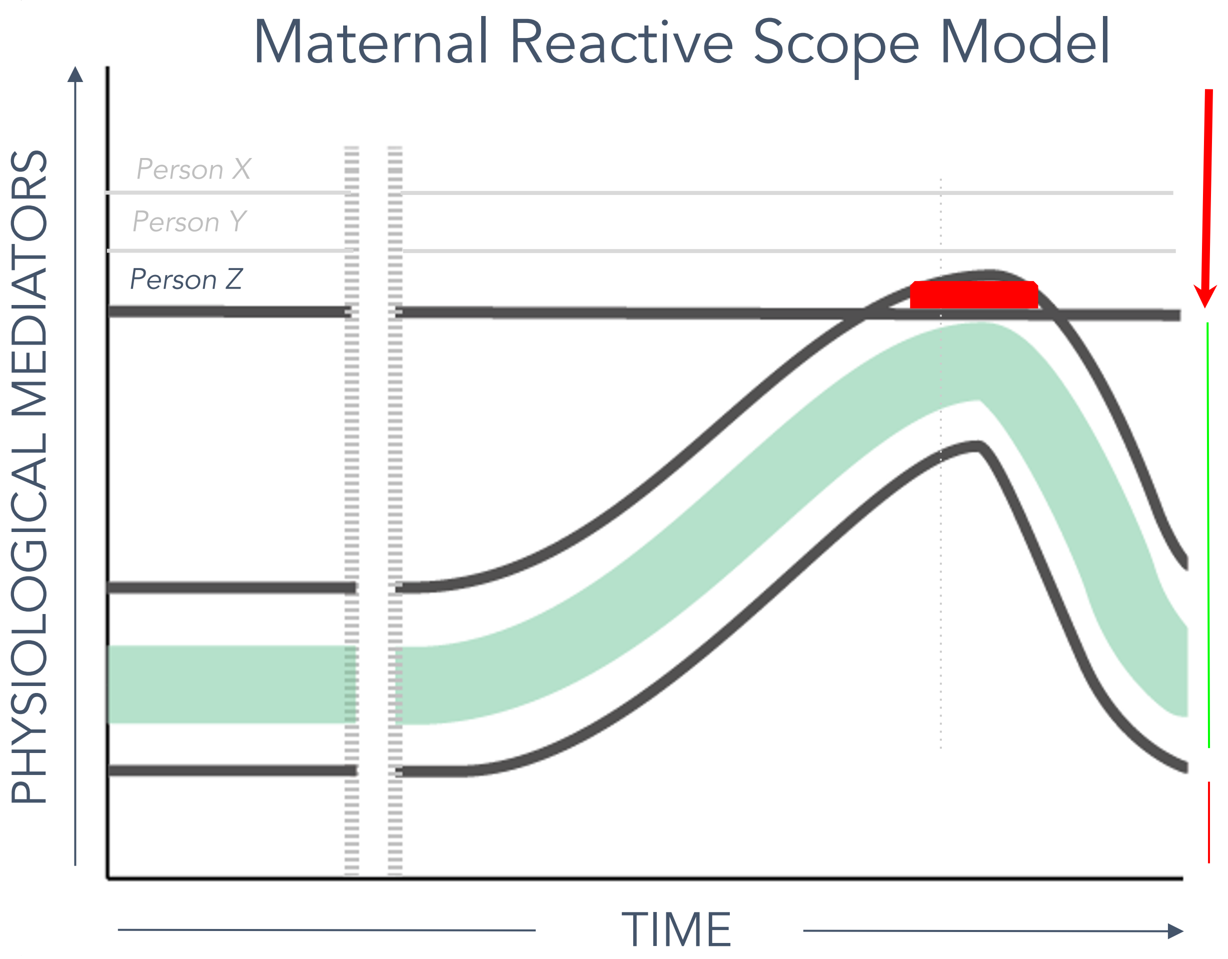

From: Dickens MJ, Pawluski JL and Romero LM (2020) Moving Forward From COVID-19: Bridging Knowledge Gaps in Maternal Health With a New Conceptual Model. Front. Glob. Womens Health 1:586697. doi: 10.3389/fgwh.2020.586697

Individual differences may increase risk of *Homeostatic Overload:*

- **Genetic susceptibility**

*For some individuals, no matter how healthy and stress-free they remain during pregnancy, **the natural physiological changes of pregnancy alone will lead to health complications***

(e.g. genetic susceptibility to mental illness linked with perinatal depression)

Upper threshold may be lower *prior to pregnancy*...

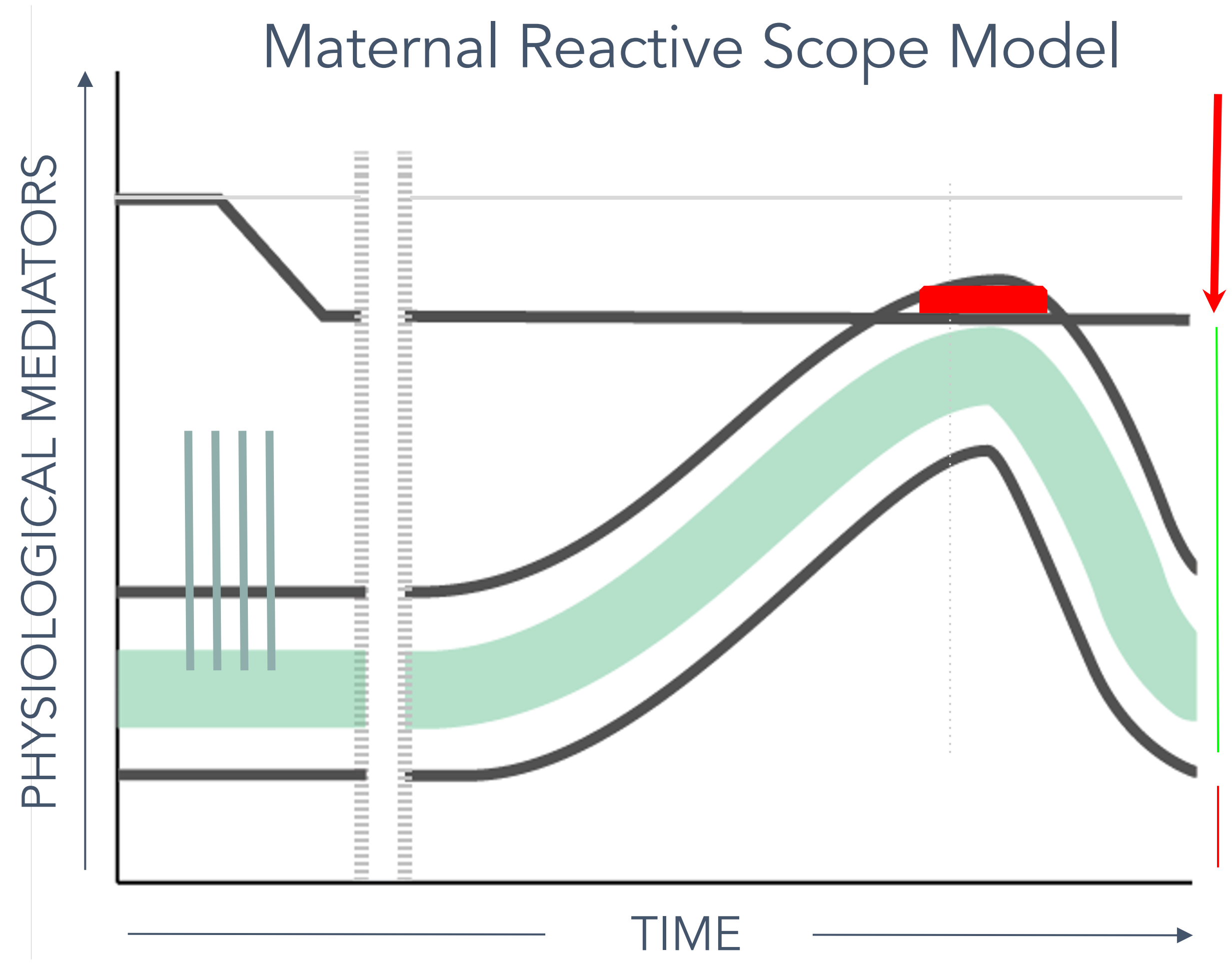

From: Dickens MJ, Pawluski JL and Romero LM (2020) Moving Forward From COVID-19: Bridging Knowledge Gaps in Maternal Health With a New Conceptual Model. Front. Glob. Womens Health 1:586697. doi: 10.3389/fgwh.2020.586697

Individual differences may increase risk of *Homeostatic Overload:*

- Genetic susceptibility
- Chronic life stress prior to pregnancy

Stress exposure prior to gestation can lead to long-lasting reduction in upper threshold and make individuals more vulnerable to illness and complications during pregnancy.

(e.g. stress of institutionalized racism underlying racial disparities in maternal and infant mortality and morbidity.

This is a *simplified starting point...*

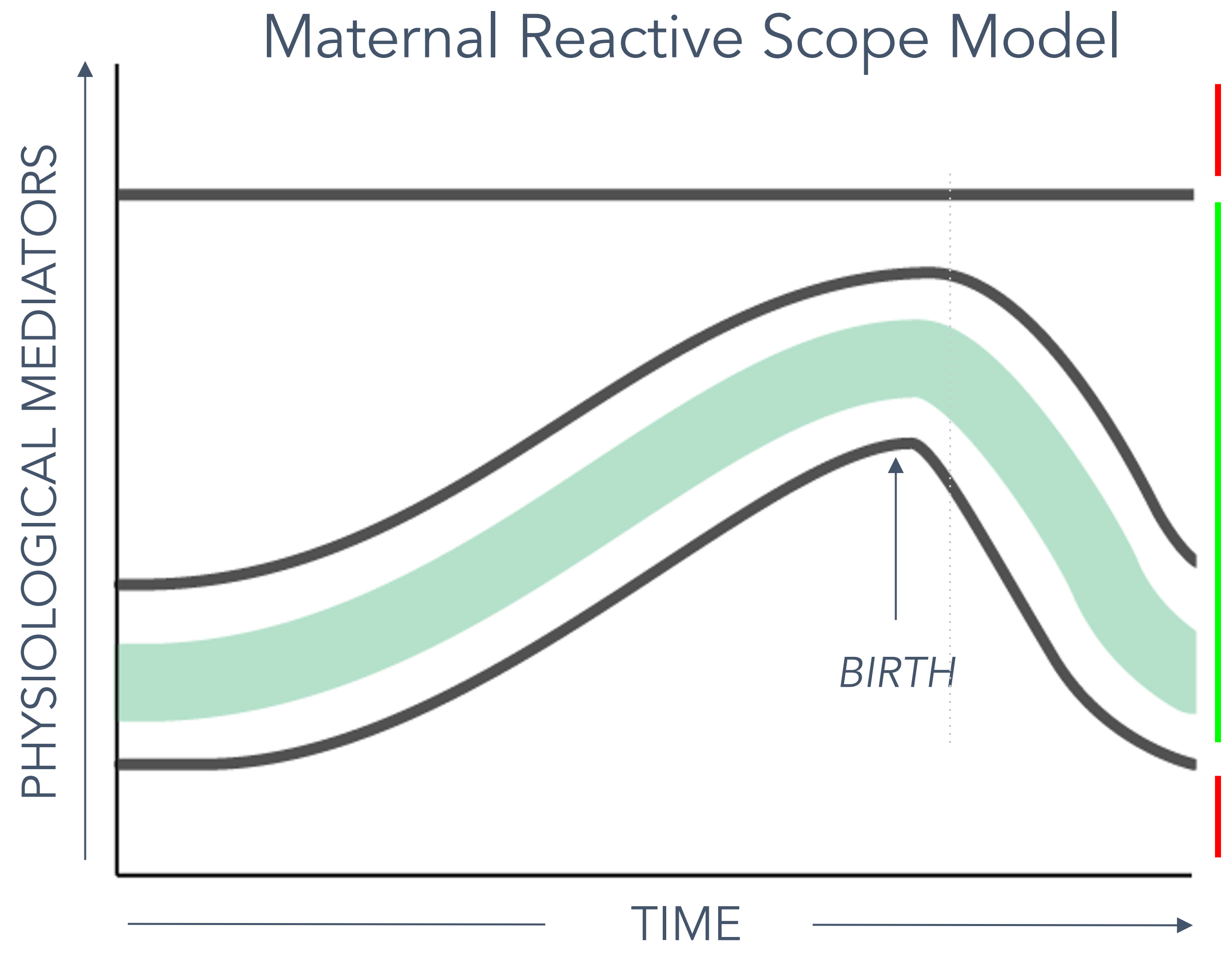

From: Dickens MJ, Pawluski JL and Romero LM (2020) Moving Forward From COVID-19: Bridging Knowledge Gaps in Maternal Health With a New Conceptual Model. Front. Glob. Womens Health 1:586697. doi: 10.3389/fgwh.2020.586697

There is nuance and many possible permutations.

For example:

- Lines, angles, rates of change/recovery may depend on individual and/or physiological mediator
- Postpartum levels and recovery may look completely different with more research and individual differences (e.g. breastfeeding)

Now what?

From: *Dickens MJ, Pawluski JL and Romero LM (2020) Moving Forward From COVID-19: Bridging Knowledge Gaps in Maternal Health With a New Conceptual Model. Front. Glob. Womens Health 1:586697. doi: 10.3389/fgwh.2020.586697*

The Maternal Reactive Scope Model is intended to present an intellectually satisfying *merge* between *"natural"* and *"medical"* approaches to pregnancy -- **healthy physiological changes during pregnancy operate *alongside* increased susceptibility to pathology.**

Another intention is to provide a new conceptual model to consider the role of stress during the perinatal period:

- Stress factors into mom's health during and after pregnancy.
- Limiting stress and/or understanding impact of stress on mother's health and health of maternal-fetal unit could factor into care pathways -- ***stress can exacerbate, stress-reduction can alleviate.***
- Considering links between **stress and maternal health** may allow for personalized care at individual and community level.

From: Dickens MJ, Pawluski JL and Romero LM (2020) Moving Forward From COVID-19: Bridging Knowledge Gaps in Maternal Health With a New Conceptual Model. Front. Glob. Womens Health 1:586697. doi: 10.3389/fgwh.2020.586697

For example:
STRESS could tip the scales for certain women…

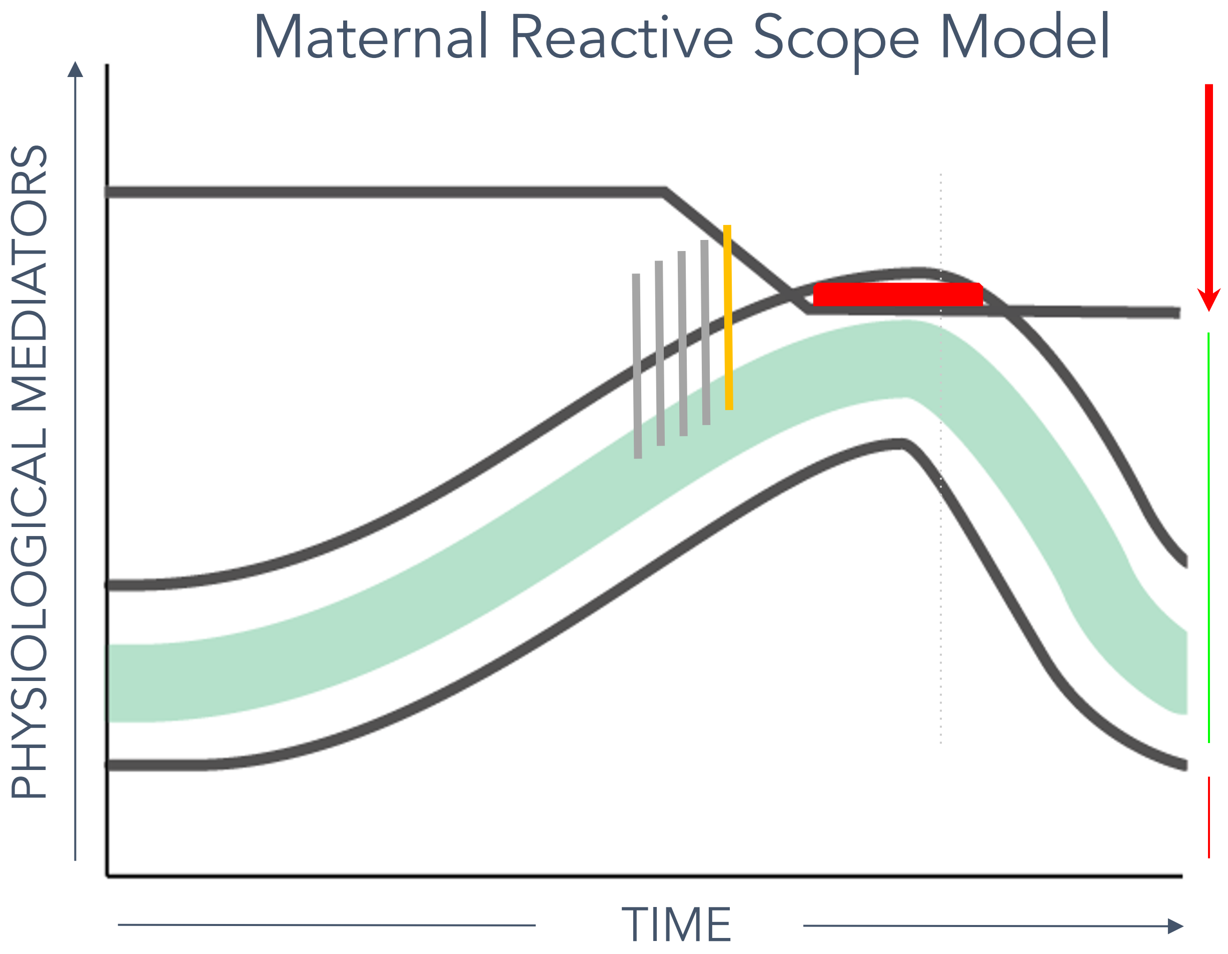

From: Dickens MJ, Pawluski JL and Romero LM (2020) Moving Forward From COVID-19: Bridging Knowledge Gaps in Maternal Health With a New Conceptual Model. Front. Glob. Womens Health 1:586697. doi: 10.3389/fgwh.2020.586697

..especially during the window of increased vulnerability.

*Thinking with this framework allows for a **different approach to predicting or preventing maternal mental health challenges** that are currently limited by a limited understanding of physiological mechanisms.*

For example:
STRESS could tip the scales for certain women...

Maternal Reactive Scope Model

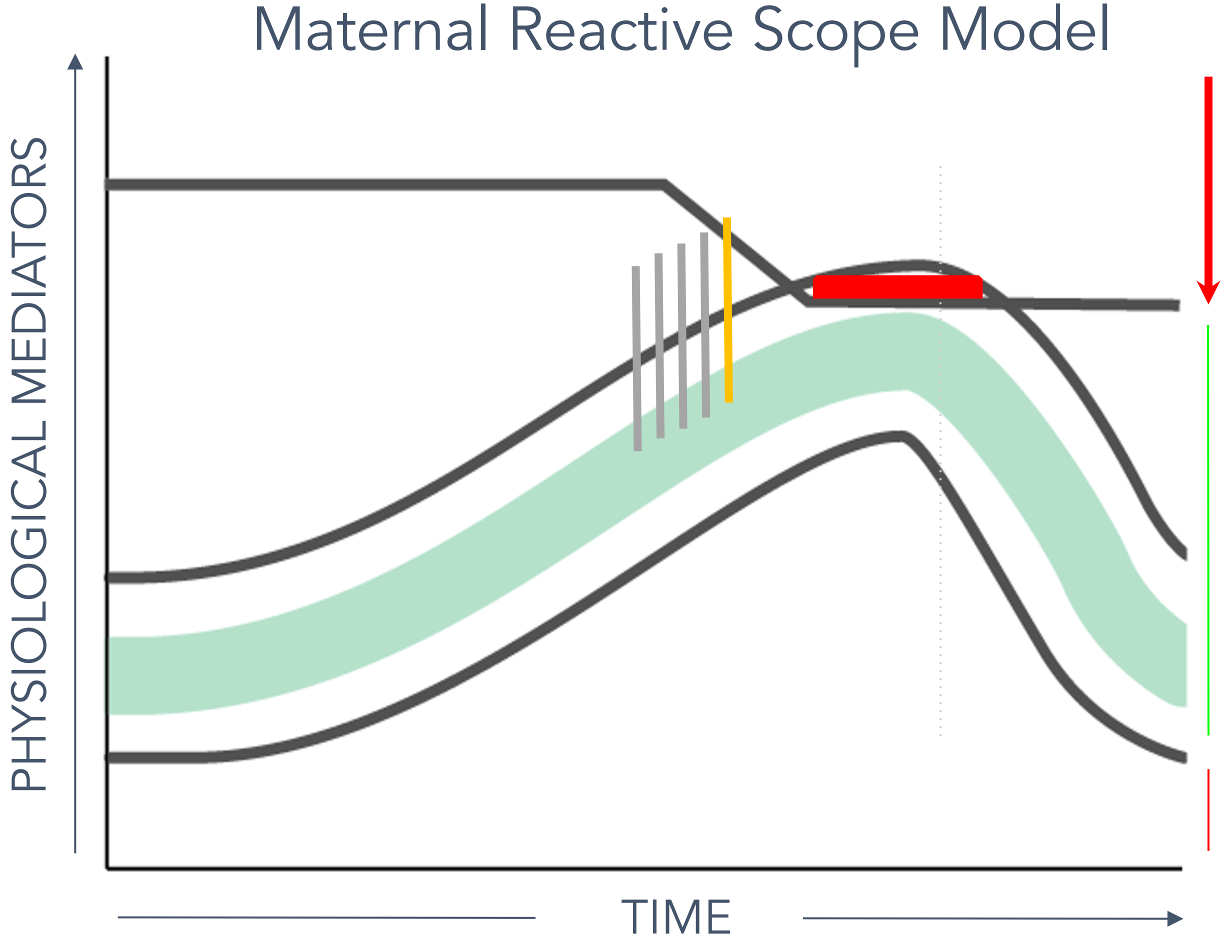

From: Dickens MJ, Pawluski JL and Romero LM (2020) Moving Forward From COVID-19: Bridging Knowledge Gaps in Maternal Health With a New Conceptual Model. Front. Glob. Womens Health 1:586697. doi: 10.3389/fgwh.2020.586697

...especially during the window of increased vulnerability.

How can we prevent stress before, during and after pregnancy?

Let's go back to this slide.

Some things to keep in mind…

A stress response is (most often) the ***physiological manifestation*** of a ***psychological input.***

- If you don't *perceive* the input as a stressor, you won't *mount* a stress response.

Not all stress responses are created equal.

- Different pathways regulate ***how much*** you physiologically respond to a stressor.
  - Physiological changes/differences can affect how *your body* physically responds to a specific stressor.
- The physiological response can be moderated.
  - Individual/contextual differences in ***perception of the severity*** of the stressor determine ***your*** individual response.
  - It's not all or nothing -- For example, you can mount a Fight-or-Flight response without a cortisol response.

From: Dickens MJ, Pawluski JL and Romero LM (2020) Moving Forward From COVID-19: Bridging Knowledge Gaps in Maternal Health With a New Conceptual Model. Front. Glob. Womens Health 1:586697. doi: 10.3389/fgwh.2020.586697

What causes *STRESS*?

Based on decades of stress research, we know that three main types of stimulus underlie initiation of a stress response:

- Lack of control
- Unpredictability
- Novelty

Yes, these are all things that are inherent to pregnancy, labor, birth, postpartum, and life with a new baby.

From: Dickens MJ, Pawluski JL and Romero LM (2020) Moving Forward From COVID-19: Bridging Knowledge Gaps in Maternal Health With a New Conceptual Model. Front. Glob. Womens Health 1:586697. doi: 10.3389/fgwh.2020.586697

What causes *STRESS*?

Cause of stress	For example,
Lack of control	I don't feel connected to my body/baby<br>Decisions will be made for me.<br>Not the birth I expected/birth trauma<br>How is my baby doing?
Unpredictability	When will baby arrive?<br>How will labor go?<br>Fear of labor<br>Unexpected intervention
Novelty	New sensations in pregnancy - contractions vs. other things.<br>Who is in room during labor (doctor roulette, nursing staff etc.)<br>New baby - lactation, sleep, etc.

Stress is inherent to pregnancy, labor, birth, postpartum, and life with a new baby,

From: Dickens MJ, Pawluski JL and Romero LM (2020) Moving Forward From COVID-19: Bridging Knowledge Gaps in Maternal Health With a New Conceptual Model. Front. Glob. Womens Health 1:586697. doi: 10.3389/fgwh.2020.586697

What causes *STRESS*?

Cause of stress	For example,
Lack of control	I don't feel connected to my body/baby<br>Decisions will be made for me.<br>Not the birth I expected/birth trauma<br>How is my baby doing?
Unpredictability	When will baby arrive?<br>How will labor go?<br>Fear of labor<br>Unexpected intervention
Novelty	New sensations in pregnancy - contractions vs. other things.<br>Who is in room during labor (doctor roulette, nursing staff etc.)<br>New baby - lactation, sleep, etc.

Stress is inherent to pregnancy, labor, birth, postpartum, and life with a new baby,

BUT understanding what causes or limits stress may be key to reducing risk of complications

From: Dickens MJ, Pawluski JL and Romero LM (2020) Moving Forward From COVID-19: Bridging Knowledge Gaps in Maternal Health With a New Conceptual Model. Front. Glob. Womens Health 1:586697. doi: 10.3389/fgwh.2020.586697

What causes *STRESS*?

Cause of stress	For example,	Demonstrated positive/negative impacts
Lack of control	I don't feel connected to my body/baby<br>Decisions will be made for me.<br>Not the birth I expected/birth trauma<br>How is my baby doing?	Labor support (doulas, etc)<br>Mindful birthing education<br>Meditation<br>C-section
Unpredictability	When will baby arrive?<br>How will labor go?<br>Fear of labor<br>Unexpected intervention	Community support<br>Education<br>Labor support (doulas, etc)<br>Mindful birthing
Novelty	New sensations in pregnancy - contractions vs. other things.<br>Who is in room during labor (doctor roulette, nursing staff etc.)<br>New baby - lactation, sleep, etc.	Labor support (doulas, etc)<br>"Difficult" baby/community support<br>Partner support<br>Sleep disturbance

Research demonstrating links between positive or negative health outcomes (e.g. perinatal mental health) and specific interventions, external assistance, or baby-related stress/stress-relief may act through stress pathways

From: Dickens MJ, Pawluski JL and Romero LM (2020) Moving Forward From COVID-19: Bridging Knowledge Gaps in Maternal Health With a New Conceptual Model. Front. Glob. Womens Health 1:586697. doi: 10.3389/fgwh.2020.586697

CASE STUDY: applying the Maternal Reactive Scope Model to maternal brain plasticity and perinatal mental illness

- Increased brain plasticity with the transition to motherhood is normal and is linked to the rapid onset of maternal care of offspring.
- The maternal brain shows rather 'extreme' levels of plasticity, speculated to be far greater than at other times in a woman's life but still within the normal maternal reactive scope during this time.
  - This 'extreme' brain plasticity is healthy and important, not only for bonding to the neonate, but for other aspects of maternal care such as the onset of lactation
- The model predicts a disease state if the normal plasticity exceeds the upper threshold to Homeostatic Overload
  - E.g. the amygdala, an important relay center for communication-related to maternal behaviors undergoes extreme stimulation in response to stress cues (e.g. with inter partner violence or a traumatic birth). Such stimulation could push changes in the amygdala into Homeostatic Overload range -- changing neurocircuitry and leading to postpartum depression or postpartum post-traumatic stress disorder.
- Maternal Reactive Scope Model could connect pandemic-related stress – additional birth trauma, lack of social support, added financial pressure – with reported increases in maternal mental illness.

Oatridge A, Holdcroft A, Saeed N, Hajnal JV, Puri BK, Fusi L, et al. Change in brain size during and after pregnancy: study in healthy women and women with preeclampsia. AJNR Am J Neuroradiol. 52 2002;23: 19–26.
Hoekzema E, Barba-Mu□ller E, Pozzobon C, Picado M, Lucco F, Garci□a-Garci□a D, et al. Pregnancy leads to long-lasting changes in human brain structure. Nat Neurosci. 2017;20: 287–296.
Hoekzema E, Tamnes CK, Berns P, Barba-Mu□ller E, Pozzobon C, Picado M, et al. Becoming a mother entails anatomical changes in the ventral striatum of the human brain that facilitate its responsiveness to offspring cues. Psychoneuroendocrinology. 2020;112: 104507.
Slattery DA, Neumann ID. No stress please! Mechanisms of stress hyporesponsiveness of the maternal brain. J Physiol. 2008;586: 377–385.
Feldman R. The adaptive human parental brain: implications for children's social development. Trends Neurosci. 2015;38: 387–399.
Pawluski JL, Lonstein JS, Fleming AS. The Neurobiology of Postpartum Anxiety and Depression. Trends Neurosci. 2017;40: 106–120.
Roos A, Fouche J-P, Stein DJ. Brain network connectivity in women exposed to intimate partner violence: a graph theory analysis study. Brain Imaging Behav. 2017;11: 1629–1639.

From: Dickens MJ, Pawluski JL and Romero LM (2020) Moving Forward From COVID-19: Bridging Knowledge Gaps in Maternal Health With a New Conceptual Model. Front. Glob. Womens Health 1:586697. doi: 10.3389/fgwh.2020.586697

Appendix

From: Dickens MJ, Pawluski JL and Romero LM (2020) Moving Forward From COVID-19: Bridging Knowledge Gaps in Maternal Health With a New Conceptual Model. Front. Glob. Womens Health 1:586697. doi: 10.3389/fgwh.2020.586697

<u>Examples</u> of key physiological mediators

+ expected perinatal shift in levels and/or the role they play in Predictive and Reactive Homeostasis during the perinatal period.

+ potential health complications for mother, baby, or the maternal<>fetal unit when pushed beyond the upper limit (Homeostatic Overload) or fail to reach lower limit (Homeostatic Failure)

NOTE: While this is not a comprehensive list and many links between mediators and complications have been suggested but are not well understood (*), the suggested physiological mechanisms and links presented here are intended to be used to generate further discussion of the Maternal Reactive Scope Model and its potential application.

Physiological system	Physiological mediators	Peripartum Predictive/Reactive Homeostasis -	Peripartum Homeostatic Overload and/or Homeostatic Failure range
**Immune**	• Prostaglandin<br>• T-cell activation<br>• Antibody titers<br>• Cytokines	• Pro-inflammatory phases (Support implantation, Parturition)<br>• Anti-inflammatory phase (Maintenance of pregnancy)<br>• Maternal-fetal-placental interactions	• Autoimmune disease<br>• Sensitivity to infectious disease<br>• Preterm birth*<br>• Miscarriage*
**Endocrine**	• HPA<br>○ Glucocorticoids (e.g. cortisol)<br>○ CRH<br>○ Placental CRH (pCRH)<br>○ ACTH<br>• Thyroid<br>• Reproductive<br>• Progesterone<br>• Estrogens<br>• Insulin<br>• Oxytocin<br>• Melatonin<br>• Prolactin	• Cortisol increases 30x nonpregnant concentrations<br>• pCRH becomes dominant driver of maternal HPA<br>• Maternal CRH decreases<br>• HPA responsiveness decreases<br><br>• Progesterone increases nearly 10x nonpregnant concentrations<br>• Estrogens increase nearly 100x nonpregnant concentrations<br><br>• Insulin secretion increases 200% 250%<br>• Insulin sensitivity decreases up to 50%	• Perinatal mental illness*<br>• Maladaptive fetal HPA development<br><br>• Preterm birth*<br>• Miscarriage<br><br>• Insulin resistance<br>• Gestational diabetes mellitus<br>• Preeclampsia<br>• High or low birth weight
**Cardiovascular (catecholamines)**	• Cardiac output<br>• Stroke volume<br>• Heart rate<br>• Blood pressure<br>• Heart rate variability	• Cardiac output increases 30-50%<br>• Stroke volume increases up to 85mL (20 weeks gestation)<br>• Heart rate increased (up to 90-100 beats/min)<br>• Systemic vascular resistance decreased by 21% (lowest at 20-24 weeks)<br>• Pulmonary vascular resistance decreased by 34%	• Myocardial infarction<br>• Cardiac muscle breakdown<br>• Hypertension<br>• Preeclampsia
**Hematologic and coagulation systems**	• White blood cells (WBC)<br>• Red blood cells (RBC)<br>• Erythropoietin<br>• Clotting factors<br>• Fibrinogen	• RBC & WBC counts increase<br>• 30% increase in RBC mass<br>• ~45% increase in plasma volume<br>• Increased erythropoietin production<br>• Hemodilution<br>• Hypercoagulable state	• Anemia<br>• Thromboembolism
**Central nervous system**	• Neurogenesis<br>• Neurotransmitter concentrations<br>• Cytokines<br>• Neuroendocrine (e.g. Oxytocin)<br>• Neurobehavioral	• Heightened plasticity/malleability of the maternal brain<br>• Increased Oxytocin (maternal bonding)	• Depression<br>• Anxiety<br>• Post-traumatic stress disorder<br>• Attachment disorder

From: Dickens MJ, Pawluski JL and Romero LM (2020) Moving Forward From COVID-19: Bridging Knowledge Gaps in Maternal Health With a New Conceptual Model. Front. Glob. Womens Health 1:586697. doi: 10.3389/fgwh.2020.586697
